# Chromosome-level genome and multi-omics analyses provide insights into the geo-herbalism properties of *Alpinia oxyphylla*

**DOI:** 10.3389/fpls.2023.1161257

**Published:** 2023-06-08

**Authors:** Kun Pan, Shuiping Dai, Jianping Tian, Junqing Zhang, Jiaqi Liu, Ming Li, Shanshan Li, Shengkui Zhang, Bingmiao Gao

**Affiliations:** ^1^ Hainan Provincial Key Laboratory for Research and Development of Tropical Herbs, Haikou Key Laboratory of Li Nationality Medicine, Hainan Ouality Monitoring and Technology Service Center for Chinese Materia MedicaRaw Materials, School of Pharmacy, Hainan Medical University, Haikou, Hainan, China; ^2^ Academician Workstation of Hainan Province and The Specific Research Fund of The Innovation Platform for Academicians of Hainan Province, Haikou, Hainan, China; ^3^ School of Bioengineering, Qilu University of Technology (Shandong Academy of Sciences), Jinan, Shandong, China

**Keywords:** *Alpinia oxyphylla*, genome, metabolomics, geo-herbalism, transcriptomics, nootkatone, valenene synthase

## Abstract

**Introduction:**

*Alpinia oxyphylla* Miquel (*A. oxyphylla*), one of the “Four Famous South Medicines” in China, is an essential understory cash crop that is planted widely in the Hainan, Guangdong, Guangxi, and Fujian provinces. Particularly, *A. oxyphylla* from Hainan province is highly valued as the best national product for geo-herbalism and is an important indicator of traditional Chinese medicine efficacy. However, the molecular mechanism underlying the formation of its quality remains unspecified.

**Methods:**

To this end, we employed a multi-omics approach to investigate the authentic quality formation of *A. oxyphylla*.

**Results:**

In this study, we present a high-quality chromosome-level genome assembly of *A. oxyphylla*, with contig N50 of 76.96 Mb and a size of approximately 2.08Gb. A total of 38,178 genes were annotated, and the long terminal repeats were found to have a high frequency of 61.70%. Phylogenetic analysis demonstrated a recent whole-genome duplication event (WGD), which occurred before *A. oxyphylla’s* divergence from W. villosa (~14 Mya) and is shared by other species from the Zingiberaceae family (Ks, ~0.3; 4DTv, ~0.125). Further, 17 regions from four provinces were comprehensively assessed for their metabolite content, and the quality of these four regions varied significantly. Finally, genomic, metabolic, and transcriptomic analyses undertaken on these regions revealed that the content of nootkatone in Hainan was significantly different from that in other provinces.

**Discussion:**

Overall, our findings provide novel insights into germplasm conservation, geo-herbalism evaluation, and functional genomic research for the medicinal plant *A. oxyphylla*.

## Introduction

Zingiberaceae is a large, fragrant pantropical family consisting of 1,600 species divided among about 50 genera (Christenhusz and Byng, 2016). The plants are distributed throughout tropical Africa, Asia, and the Americas (Ra Mans et al., 2019). Zingiberaceae plants contain many bioactive terpenoids, flavonoids, and polyphenols that are economically important as traditional medicines, spices, and cosmetics. *Alpinia oxyphylla* Miquel (*A. oxyphylla*) is one of the type species in the genus *Alpinia* (with more than 230 species) and has been approved by China Food and Drug Administration as a medicine and food homology species. Additionally, *A. oxyphylla* has been used as a medicinal and edible plant for hundreds of years. Its fruit when dried or baked with salt is referred to as Fructus Alpiniae Oxyphyllae (FAO), and the Chinese medicine name is “Yi Zhi, Yi Zhi Ren”—in traditional Chinese medicine (TCM), it is one of the “Four Famous South Medicines”. FAO is commonly used as a medicine for warming the kidney and spleen, securing essence and arresting polyuria, and stopping diarrhea and saliva in TCM. Classified Materia Medica (1097 A.D.-1108 A.D.) and the Compendium of Materia Medica (1552 A.D.-1578 A.D.) document the use of FAO either alone or in combination with other herbal medicines. Various pharmacological properties of FAO have been reported, such as anti-inflammatory (Yu et al., 2020), anti-oxidant (Thapa et al., 2021), anti-diarrheal (Zhang et al., 2013), anti-Alzheimer’s disease (Li et al., 2021b), promoting neuronal regeneration and resisting neurodegenerative diseases (He et al., 2018), and anti-diuretic and diuretic (Li et al., 2016). The unique medicinal and flavor characteristics of *A. oxyphylla* are associated with a variety of metabolites, including rich terpenoids (Chen et al., 2014), diarylheptanoids (Chen et al., 2014), and diarrhea (Zhang et al., 2013). However, there have been some reports on the identification and functional characterization of genes related to the biosynthesis of flavonoids (Yuan et al., 2021) and terpenoids (Yang et al., 2022) in Zingiberaceae. There are multiple kinds of terpenoids, diarylheptanes, and flavonoids in *A. oxyphylla*, and the biosynthetic pathway remains largely unexplored.

*A. oxyphylla* is a kind of herbaceous plant, which thrives in tropical rain forest and evergreen broad-leaved forests. It is commonly cultivated under rubber forest, pine forest, and eucalyptus forest in the Hainan, Guangdong, Guangxi, and Fujian provinces. Our study on various *A. oxyphylla* cultivation regions reveals that the main components vary depending on the region. Biological properties of medicinal plants are highly influenced by the environment; thus, the chemical composition and content of medicinal plants are dependent on their environment (Ma et al., 2010; Ma et al., 2018; Li et al., 2020b). Traditional Chinese medicinal philosophy only recognizes and values geo-herbs as authentic medicines, and only these are considered safe and of high quality (Chen et al., 2017).

Our previous study conducted an investigation on the wild and cultivated populations of *A. oxyphylla* from various geographical locations and discovered that the individual genetic diversity of *A. oxyphylla* is significantly high (Wang et al., 2012). Despite observing instances of inbreeding and gene flow (Nm=1.453) in *A. oxyphylla* populations, the phylogenetic relationship among certain accessions remains relatively distant due to the notable genetic differentiation of the wild population compared to that of the cultivated population. Additionally, the grouping of all accessions almost completely aligns with their geographical origin, demonstrating the evident regional differentiation of this species (Zou et al., 2013). Furthermore, our research delved into the transcriptome and metabolome of different tissues and fruit development stages of *A. oxyphylla* and identified differentially expressed genes associated with the biosynthesis of flavonoids (Yuan et al., 2021) and terpenoids (Pan et al., 2022), highlighting the primary medicinal component, nootkatone, which primarily significantly accumulates in seeds during the late stage of development. Unfortunately, the lack of genomic data has obstructed a proper attribution of these compounds to specific genes in the biosynthetic pathway. However, with the recent completion of genome sequencing in several Zingiberaceae plants, employing metabonomics, transcription, and genome association methods to analyze the main active components and related biosynthetic pathways has become crucial. Thus, obtaining a comprehensive understanding of the genetic structure of *A. oxyphylla* is pivotal in order to furnish a basis for geo-herbalism evaluation in different planting areas.

Currently, numerous species have seen completed molecular markers, gene mining and cloning, and the functional identification of important agronomic traits, marking the onset of the post-genome era. However, research on Zingiberaceae is still in its nascent stages, with only a few genomes having been sequenced, including those of *Amomum tsao-ko* (Li et al., 2022), *Zingiber officinale* (Cheng et al., 2021b; Li et al., 2021a), *Wurfbainia villosa* (Yang et al., 2022), *Curcuma alismatifolia* (Liao et al., 2022), *Curcuma longa* (Chakraborty et al., 2021), and *Alpinia nigra* (Ranavat et al., 2021). It is worth noting that the genomes of *Curcuma longa* and *Alpinia nigra* are only a sketch, meaning that the whole-genome information of genus *Alpinia* has yet to be revealed or reported. Obtaining a high-quality genome will provide sufficient data for solving phenotypic and genetic variations, advancing studies on the molecular basis of characteristic metabolites in *A. oxyphylla*, and guiding breeding strategies aimed at improving characteristic components.

The present study assembled a high-quality genome, transcriptome, and metabolism of *A. oxyphylla*. It has also revealed that nootkatone can be employed as an indicator to identify the *A. oxyphylla*’s geo-herbalism. The expansion of the valencene synthase gene family is regarded as being responsible for the regional variation of nootkatone content. These findings offer insights into molecular breeding and functional gene identification related to important traits of *A. oxyphylla*. Furthermore, the high-quality reference genome of *A. oxyphylla* presented in this study provides a valuable resource for exploring the evolution, speciation, and geo-herbalism of other species in the Zingiberaceae family.

## Materials and methods

### Plant materials

**Figure 1:**
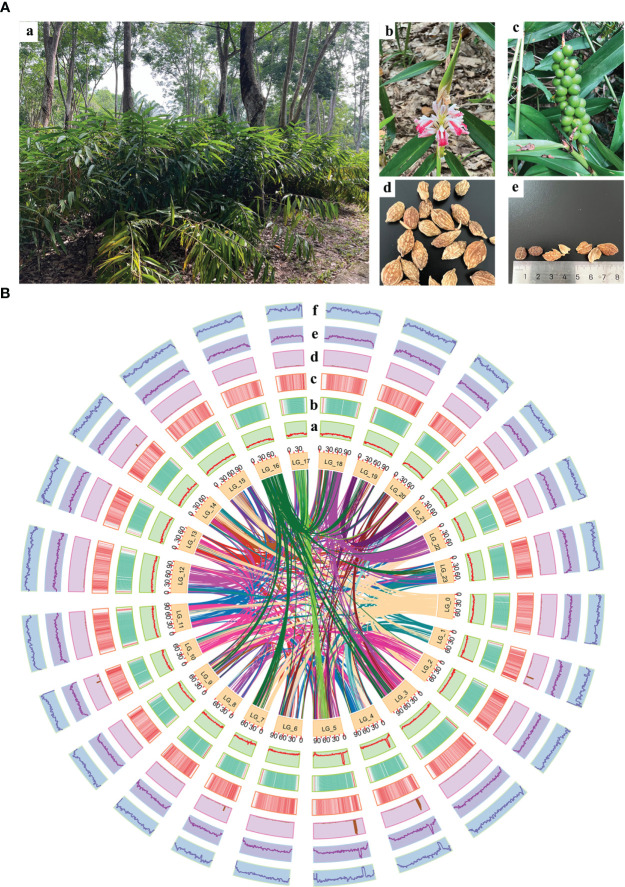
Morphology of *A*. *oxyphylla* and overview of *A*. *oxyphylla* gnome. **(A)** Morphological characteristics of *A. oxyphylla*. (a), Plant; (b), inflorescence; (c), fruits; (d), dried fruit; (e) seed and pericarp. **(B)** Circos plot of *A. oxyphylla* genome assembly. Elements are arranged in the following scheme (from inner to outer): (a) GC content; (b) gene density shown as the distribution densities from high (red) to low (green); (c) repeat sequence densities; (d) non-coding RNA (ncRNA) density; (e) tandem repeat density; (f) the density of other repeats except tandem repeats. The window size is 1Mb.

The plant materials were collected from a cultivar of “changyuanguo” from Danzhou (19°51′N, 109°50′E), Hainan Province, China, which is considered the authentic production area of *A. oxyphylla* (**Figure 1A**). Its leaves were used for genomic sequencing, and its roots were used for flow cytometry and karyomorphological analysis. The fruit utilized for transcriptome and metabolome analysis consisted of an oval-shaped variety and a more commonly found and higher yielding type. A total of three and six biological replicates, respectively, were gathered from Baoting, Danzhou, Naning, and Zhangpu during May of 2019, as outlined in **Supplementary Table 1**. Tissue from the fruit was uniformly collected at 55-65 days after fruiting, promptly frozen in liquid nitrogen, and stored at -80°C.

### Somatic chromosome numbers and karyomorphological analysis

We used living tissue samples from the root tips of *A. oxyphylla* and promoted cell cycle synchronization as needed. The cell mitosis was fixed in the metaphase phase, and then the cellulose enzyme process was used to obtain mid-mitotic division phase. Finally, we performed 4’,6-diamidino-2-phenylindole (DAPI) staining and capture under a fluorescence microscope (Leica DM2500) to avoid light microscopy.

Twenty scattered cells with good morphology were selected from each material for chromosome counting, and five metaphone cells with clear chromosome morphology and no overlap were used for karyotype analysis. After measuring its length, the homologous pairing was carried out according to the morphological characteristics of chromosomes and the analysis of the measured data, and the karyotype map was arranged (**Supplementary Table 2**).

### Genome size and heterozygosity estimation

After nuclear extraction and staining, the nucleus of *A. oxyphylla* was prepared to for measurement by flow cytometry. *Manihot esculenta* and *Solanum lycopersicum*, with their known genome size, were selected as internal reference species. CFlow Plus 1.0.264.15 software was used for data collection.

To determine the genome characteristics of *A. oxyphylla*, K-mer (k = 17), analysis was performed on the Illumina Hiseq platform with the insert size of 350bp. SOAPnuke (V1.6.5) was used to filter raw sequencing data. The software “kmer_freq_stat”, independently developed by Biomarker Technologies Co., Ltd, was used to calculated the depth distribution map of each K-mer, and the heterozygosity rate was calculated according to Marcais and Kingsford (2011).

### Library construction and sequencing

Young leaf samples were collected in May 2020, and their total genomic DNA was extracted using the DNAsecure Plant Kit (TIANGEN). The quality of isolated genomic DNA was verified by electrophoresis and the Qubit dsDNA hs assay kit in Qubit ^®^3.0 Flurometer (Life technologies, AC, USA); 0.3μg DNA per sample was used for library generation. Fragments (350 bp) were generated and used to construct a sequencing library. At last, 150-bp paired-end reads were used to sequence on the Truseq Nano DNA HT Sample Prep Kit (Illumine HiSeq X-Ten platform, USA).

The circular consensus sequencing (CCS) approach was selected for single-molecule real-time (SMRT) long-read sequencing. Five 20-kb insert libraries were prepared using SMRTbell Express Template Prep Kit 2.0e, and a total of nine SMRT cells with 80.10 Gb of sequence data (54-fold coverage of the genome) were obtained and sequenced on the PacBio Sequel II platform.

For Hi-C sequencing, we used 1% formaldehyde solution in an MS buffer (50 mM NaCl; 10 mM potassium phosphate, pH 7.0; 0.1M sucrose) to fix fresh leaves at room temperature for 30 min in a vacuum. After fixation, the leaves were incubated under a vacuum in the MC buffer and then resuspended in a nuclei isolation buffer and filtered. Chromatin extraction and DNA were digested by the HindIII restriction enzyme (NEB), and then they were labeled with biotin on the DNA ends and incubated. Proteinase K was added to reverse cross-linking before ligation. After removing the unligated ends, the purified DNA was sheared to a size of 300-500 bp fragments, and we repaired the DNA ends. Then, the separated DNA fragments were labeled by biotin with Dynabeads^®^ M-280 Streptavidin (Life Technologies). We used the Illumina Hiseq X Ten sequencer to control the Hi-C libraries quality and sequence them.

In addition, the total RNA of the same *A. oxyphylla* individual was extracted from seven tissues (root, stem, leaf, fruit, seed, pericarp, and suction bud) with three biological replicates, and its RNA was extracted using a RNeasy plant mini kit (Qiagen). The RNA Nano 6000 Assay Kit of the Bioanalyzer 2100 system (Agilent Technologies, CA, USA) was employed to assess its integrity.

### Genome assembly and quality assessment

The *A. oxyphylla* genome was assembled as follows: firstly, after quality controlling of the raw Hi-C data using HI-C-PRO (version 2.8.0) (Servant et al., 2015), contigs were assembled from CCS clean reads with default parameters using Hifiasm (V 0.12) (Cheng et al., 2021a). Secondly, the high-quality paired-end Hi-C reads were first mapped to the reference *A. oxyphylla* genome (GRCm38/mm10) using the Burrows-Wheeler Aligner (BWA) software (Li and Durbin, 2009). We converted the alignment files to BAM files using SAMtools (Li and Durbin, 2009), and then we improved the alignment results; only uniquely alignable pairs reads (mapping quality >20) were selected for further analysis, and we filtered out low-quality sequences using FASTP (version 0.12.6) (Chen et al., 2018a; Chen et al., 2018b). The present study involved a manual inspection of segments that displayed conflicting associations with information obtained from the raw scaffold. Subsequently, a chromosome-level assembly was generated from the draft contig-level assembly by utilizing the LACHESIS34 software, which employs the ligating adjacent chromatin enables scaffolding *in situ* method (Burton et al., 2013).

The accuracy and completeness of the genome assembly were evaluated using several methods. Firstly, the Hi-C interaction heatmap was employed to determine the organization of the genome. Secondly, the presence of contamination in the sequencing data was assessed using GC depth scatter plots and GC content. Thirdly, the alignment of the genome sequencing to the assembled genome was conducted to assess the coverage. Finally, two core eukaryotic gene datasets were utilized to assess the completeness of the genome: Benchmarking Universal Single-Copy Orthologs (BUSCO), accessible at http://busco.ezlab.org/, and Core Eukaryotic Genes Mapping Approach (CEGMA), accessible at http://korflab.ucdavis.edu/datasets/cegma/.

### Genome annotation

The tandem repeat sequence was predicted ab initio using TRF (http://tandem.bu.edu/trf/trf.html). Repeat regions were extracted *via* homolog prediction by employing the Repbase (Jurka et al., 2005) database and the RepeatMasker (http://www.repeatmasker.org/) software, along with its in-house scripts (RepeatProtein Mask), using default settings. To build a *de novo* repetitive elements database, LTR_FINDER (http://tlife.fudan.edu.cn/ltr_finder/), RepeatScout (http://www.repeatmasker.org/), and RepeatModeler (http://www.repeatmasker.org/Repeat Modeler.html) were utilized with default parameters. The resultant TE library consisted of all repeat sequences longer than 100bp and with gaps “N” less than 5%. The Repbase and *de novo* TE libraries constituted a custom library supplied to RepeatMasker for identifying DNA-level repeats. Protein-coding genes were predicted using *de novo* gene prediction, homolog prediction, and RNA-seq-based prediction. For the former, ab initio-based gene prediction was performed using Augustus (v3.2.3), Geneid (v1.4), Genescan (v1.0), GlimmerHMM (v3.04), and SNAP (2013-11-29). Homologous protein sequence data were obtained from Ensembl/NCBI/others, and TblastN (v2.2.26; E-value ≤ 1e-5) was used to align the protein sequences to the genome. (Birney et al., 2004) software was then used to predict the gene structure contained in each protein region *via* accurate spliced alignments with the homologous genome sequences. Then, RNA-Seq reads from different *A. oxyphylla* tissues were mapped to the assembled genome by utilizing TopHat (v2.0.11) (Trapnell et al., 2009). Furthermore, GeneMarkS-T (v5.1) 48 was employed to predict genes based on the assembled transcripts (Tang et al., 2015). Finally, the EVM software (v1.1.1) was utilized to combine gene models from the above approaches (Haas et al., 2008).

Gene function predictions were determined by aligning protein sequences to Swiss-Prot *via* Blastp, using a threshold of E-value ≤ 1e-5 for the best match. Motifs and domains were annotated with InterProScan (v4.8) (Mulder and Apweiler, 2007) by querying against a range of publicly available databases, such as ProDom, PRINTS, Pfam, SMRT, PANTHER, and PROSITE. The corresponding InterPro entry for each gene was used to assign gene ontology (GO) IDs. Additionally, we mapped each gene set to a KEGG pathway and identified the best match for each gene.

### Phylogenetic tree construction and evolution rate estimation

To determine the phylogenetic relationships between *A. oxyphylla* and other closely related species, we utilized protein sequences from a set of 832 single-copy ortholog genes. These sequences were aligned using the mafft (v7.205) program designed by (Katoh et al. (2009) and subsequently curated with gblocks (v0.91b). The resulting coding DNA sequences (CDS) alignments were concatenated, guided by the protein alignment, and used to construct a phylogenetic tree with the aid of iqtree (v1.6.11) developed by Nguyen et al. (Nguyen et al., 2015).

### Gene family analysis

To cluster families of protein-coding genes, we analyzed proteins from the longest transcripts of each gene from *A. oxyphylla* and other nine closely related species, namely, *Arabidopsis thalian, Sorghum bicolor, Oryza sativa*, *Ananas comosus*, *Musa balbisiana*, *Musa acuminata*, *Zingiber officinale*, *Curcuma alismatifolia*, and *Wurfbainia villosa.* We used the OrthoFinder (v2.5.1) software (Emms and Kelly, 2019) to compare protein-coding sequences within the genomes of *A. oxyphylla* and the other nine species. We then annotated the obtained gene families using the Pfam V33.1 database (Mistry et al., 2021). Using the identified gene families and predicted divergence time, we constructed a phylogenetic tree of these species and analyzed gene family expansion and contraction using CAFE (Han et al., 2013). In CAFE, a random birth and death model is proposed, allowing for the study of gene gain or loss across a specified phylogenetic tree. We calculated a conditional p-value for each gene family and considered those with a conditional p-value of less than 0.05 to have an accelerated rate of gene gain or loss.

### Whole-genome duplication and the insert time of LTR calculation

The identification of whole-genome duplication events in *A. oxyphylla* was performed using the synonymous mutation rate (Ks) method and the fourfold synonymous third-codon transversion rate (4DTv) method. Initially, the software wgd (v1.1.1), developed by Zwaenepoel and Van De Peer (2019), and a custom script (https://github.com/JinfengChen/Scripts) were utilized for this purpose. The identification of full-length long terminal repeat retrotransposons (fl-LTR-RTs) was achieved through the utilization of both LTRharvest (v1.5.10) (Ellinghaus et al., 2008) and LTR_finder (v1.07) (Xu and Wang, 2007). LTR_retriever (Ou and Jiang, 2018) was then used to produce high-quality intact fl-LTR-RTs and a non-redundant LTR library. To determine the distance between the flanking sequences on both sides of LTR, mafft (v7.205) (Katoh et al., 2009) was used for comparison, and the Kimura model in EMBOSS (v6.6.0) (Rice et al., 2000) was employed for distance calculation.

### RP-HPLC analysis

After 14 days of drying at 45°C in a drying oven, the fruit was polished into powder and accurately weighed, 25mL 70% ethanol was added, then ultrasonic extraction was performed for three times, each time for 30 minutes, centrifugation was performed several times and the supernatant was taken to obtain the test product solution. Chromatographic column: Phenomenex Gemini C6-phenyl (250 mm× 4.6mm, 5μm); mobile phase: acetonitrile (A)–water (B) solution, gradient elution [0-5 min, A-B(40:60); 6-26, A-B(60:40); 27-32, A-B(40:60)]; flow rate: 1.0 ml/min^-1^; detection wavelength: 240 nm; column temperature: 30°C. The result is shown in **Supplementary Table 3-6**.

### Transcriptome sequencing and analysis

In this study, total RNA was extracted from A. oxyphylla using the RNAsecure Plant Kit (TIANGEN). The resulting RNA was assessed for purity using a NanoPhotometer^®^ spectrophotometer (IMPLEN, CA, USA) and for integrity using the RNA Nano 6000 Assay Kit on the Agilent Bioanalyzer 2100 system (Agilent Technologies, CA, USA). Sequencing libraries were generated using the NEBNext^®^ Ultra™ RNA Library Prep Kit for Illumina^®^ (NEB, USA), following the manufacturer’s recommendations, with index codes added to attribute sequences to each sample. The total RNA content used was 1.5 μg. To cluster the index-coded samples, we employed the TruSeq PE Cluster Kit v3-cBot-HS (Illumina) and followed the manufacturer’s instructions. The library preparations were subsequently sequenced using the Illumina HiSeq platform, generating paired-end reads.

### Qualitative and quantitative analysis of metabolites

The metabolites were analyzed by injecting them into an LC-MS/MS system manufactured by Thermo Fisher in the USA, as described by Bondia-Pons et al. (2013). Subsequently, the raw files obtained by mass spectrometry were processed using CompoundDiscoverer 3.1 software, also developed by Thermo Fisher Scientific. Spectrogram processing and database searches were conducted to obtain qualitative and quantitative results for the metabolites. Quality control analysis was then performed to ensure the accuracy and reliability of the data. Multivariate statistical analysis methods, such as principal component analysis (PCA) and partial least square discriminant analysis (PLS-DA), were applied to the data to identify and analyze the metabolites. Finally, the biological significance of the metabolites was established using functional analysis of metabolic pathways.

### Differential expression analysis and co-expression network analysis

The differential expression analysis of the two groups was carried out using the R package ‘DESeq’ version 1.10.1. To control the false discovery rate (FDR), the P-value was adjusted using the method of Benjamini and Hochberg (1995). Genes with an adjusted P-value of less than 0.05 were classified as differentially expressed. The R package ‘GOseq’, which utilizes the Wallenius non-central hypergeometric distribution to account for gene-length bias in DEGs, was used for GO enrichment analysis of the identified DEGs (Young et al., 2010). To test the statistical enrichment of DEGs in the KEGG pathways, the KOBAS software (Mao et al., 2005) was employed. The collected multidimensional data were subjected to regression and reduction analysis, including PCA and PLS-DA, with a focus on preserving original information to the fullest. This approach facilitated the identification of differential metabolites. Subsequently, correlation analysis between significantly altered genes from the transcriptome analysis and significantly altered metabolites from the metabolomics analysis was performed using the Pearson correlation coefficient (Pearson’s r). This measure helped quantify the degree of association between differential genes and differential metabolites. Finally, to better understand the involvement of differential genes and differential metabolites in biochemical pathways and signal transduction pathways, all the obtained differential genes and differential metabolites were simultaneously mapped onto the KEGG pathway database.

### qRT-PCR

Several genes and transcription factors (TFs) were chosen for RT-qPCR examination. The initial strand cDNA was produced through the application of the NovoScript^®^ Plus all-in-one First Strand cDNA Synthesis SuperMix (gDNA Purge, Novoprotein, Shanghai, China). The gene-specific primers are enumerated in **Supplementary Table 7**.

### Statistics and reproducibility

The data consisted of a minimum of three biological replicates. To compare different groups in pairwise fashion, statistical analysis was conducted through one-way ANOVA, which was followed by Dunnett’s test with a significance threshold of p < 0.05.

## Results

### Determination of genome size and heterozygosity

The present study conducted chromosome number measurements on the root tips of a cultivated individual of *A. oxyphylla*, which had previously been sequenced for its genome. The results revealed the karyotype formula of *A. oxyphylla* is 2n=2x=44m+2sm, displaying a relative length range falling between 6.23% and 3.15% with an asymmetry coefficient of 57.6%. The karyotype type is 1A. There exist 24 pairs of 48 chromosomes, with clear 1-2 banding on the 1st, 2nd, 3rd, 6th, and 11th chromosome pairs, including satellited chromosomes. It was indicated that *A. oxyphylla* is a homologous diploid (**Supplementary Figure 1**). The genome size is 1.79Gb, which was determined through flow cytometry, and *Manihot esculenta* and *Solanum lycopersicum* were selected as reference species (**Supplementary Figure 2** and **Supplementary Table 8**). Additionally, K-mer (Li et al., 2010) analysis was employed to evaluate the *A. oxyphylla* genome. The analysis indicated approximately 86.64 Gb of modified 17-mers, a primary peak distribution frequency appearing at depth = 40, and an estimated genome size of 2.14 Gb with 0.99% heterozygosity (**Supplementary Figure 3** and **Supplementary Table 9**). Together, these findings provide evidence that the sequenced material possesses a diploid nature, confirming the karyotype formulae 2n=2x=48 in *A. oxyphylla*, which differs from report that suggested 2n=4x=48 in the genus *Alpinia* (Saenprom et al., 2018).

### Genome sequencing, assembly, and annotation

**Figure 2:**
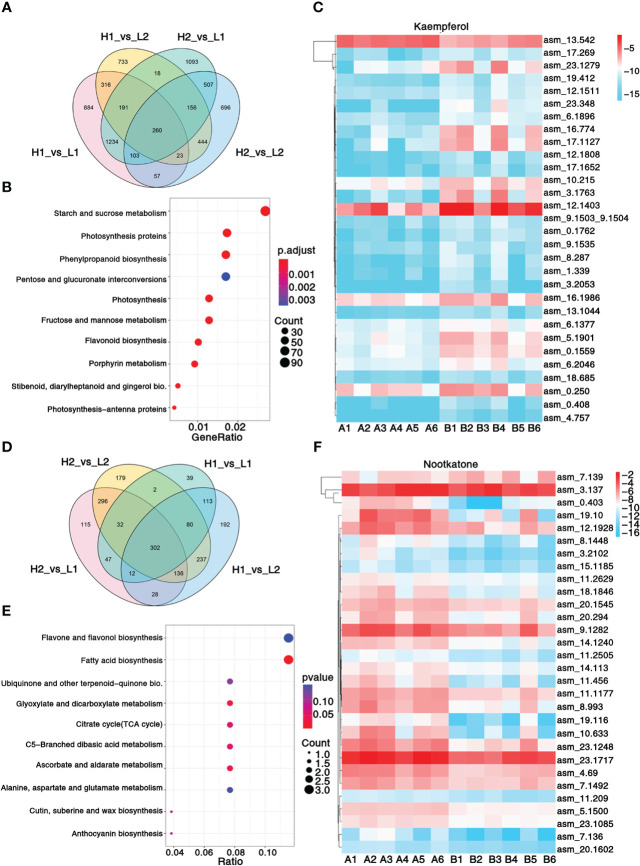
Transcriptomic and metabolic analysis and identification of differentially expressed genes. **(A)** Venn diagram shows the number of differentially expressed genes in four regions. **(B)** KEGG enrichment analysis of differentially expressed genes. **(C)** Heatmap showing the expression level of top 30 genes associated with pharmacodynamic component kaempferol. **(D)** Venn diagram shows the number of differentially expressed metabolites in four regions. **(E)** KEGG enrichment analysis of differentially metabolites. **(F)** Heatmap showing the expression level of top 30 genes associated with pharmacodynamic component nootkatone. The metabolisms KEGG annotation and correlation coefficient between genes and metabolites of this figure are from **Supplementary Data 2, 3**. Sample class: H1 (A1-A3): Danzhou; H2 (A4-A6): Baoting; L1 (B1-B3): Nanning; L2 (B4-B6): Zhangpu. The fpkm values are log2-based. Red and blue indicate high and low expression levels, respectively.

The genome of *A. oxyphylla* was sequenced using PacBio and Illumina platforms. This resulted in clean subreads of 80.10Gb with 37.35X coverage depth and clean reads of 115.85Gb with 54.02X coverage depth, as shown in **Supplementary Table 10**. Additionally, high-throughput chromosome conformation capture (Hi-C) libraries were constructed for *A. oxyphylla*, resulting in contigs totaling 2.08Gb in length, with a high contig N50 value of 76.96Mb and the longest contig of 152.6Mb. Scaffolds of 2.08Gb were collected with a scaffold N50 of 83.05Mb, with 24 scaffolds (2.00Gb) accounting for approximately 96.08% of all sequences anchored into 24 pseudochromosomes. As a result, we obtained a chromosome-level genome of *A. oxyphylla* consisting of 24 chromosomes with a total size of 2.08Gb, as indicated in **Figure 2** and **Supplementary Table 11**.

The completeness of the assembled genome of *A. oxyphylla* was evaluated using Benchmarking Universal Single-Copy Orthologs (BUSCO) and the Core Eukaryotic Genes Mapping Approach (CEGMA). Our BUSCO analysis revealed that 94.6% of the complete single-copy genes were assembled from 1614 Embryophyta-wide conserved single-copy genes. The fragmented and missing categories accounted for 1.9% and 3.5%, respectively. Additionally, CEGMA evaluation used 248 conserved genes from six eukaryotic model organisms to form a core gene library. Our evaluation showed that 235 genes were assembled with 94.76% accuracy (**Supplementary Table 12**). To further validate the accuracy of our assembly, fragments from the small fragment library were aligned to the assembled genome. Our results indicated that the alignment rate of all small reads fragments to the genome was about 99.30%, while the coverage rate was roughly 99.95%, indicating that there was a good consistency between reads and the assembled genome (**Supplementary Table 13**). Furthermore, our analysis of the heterozygous SNP ratio showed that the *A. oxyphylla* genome assembly had a high single base accuracy of 0.481%. Additionally, our distribution analysis of GC content (39.69%) and average depth confirmed that the sample was not contaminated (**Supplementary Figure 3** and **Supplementary Table 14**). Overall, these quality control metrics indicate that our *A. oxyphylla* genome assembly is complete, precise, and high quality.

In this study, a combination of homology-based searches and *de novo* annotation was employed to identify repeat sequences in *A. oxyphylla*. The total length of these sequences was found to be approximately 1.83Gb (1,836,775,398), accounting for 88.06% of the whole genome. It was observed that a large proportion of these sequences were transposable elements (TE), which constituted 87.82% of the entire genome (**Figure 1B**). The most abundant class of TE was found to be the long terminal repeats (LTR), which accounted for 61.70% of the genome. Protein-coding gene models were predicted through a combination of ab initio prediction, incorporating transcriptome, and homology (**Table 1** and **Supplementary Table 15**). A total of 38,178 protein-coding genes were predicted in *A. oxyphylla*, with an averaged gene length and CDS length of approximately 5.60Kb and 1.12Kb, respectively. Of these genes, 4,982 had homologous support, 1,229 were supported by RNA-Seq, and 593 stemmed from *de novo* gene predictions (**Supplementary Figure 5** and **Supplementary Table 16**). Then, protein sequences were predicted based on gene structure with known protein libraries, such as Swissprot, Nr, InterPro, Kyoto Encyclopedia of Genes and Genomes (KEGG), Gene Ontology (GO), and Pfam, with 75.4%, 93.9%, 54.6%, 73.7%, 81.1%, and 73.3% of these genes being functionally assigned, respectively. A total of 35,917 genes were annotated, while 2,261 genes remained unannotated. Of all the genes, 96.08% were assigned to 24 chromosomes, with a total GC content of 39.28%. These genes were unevenly distributed along the chromosomes. Most of them were focused on both ends of the chromosome, and the repetitive sequence appeared to be complementary and centromere focused (**Figure 1B** and **Supplementary Table 17**). The study also identified 534 micro RNAs (miRNAs), 3,928 tRNAs, 10,423 small nuclear RNAs (snRNAs), and 9,249 rRNAs in the *A. oxyphylla* genome (**Supplementary Table 18**).

**Table 1:** Statistics for the *A. oxyphylla* genome and gene prediction.

Assembly features	Size or number
Estimate of genome size (flow cytometry)	1.831Gb
Estimate of genome size (survey)	2.144Gb
Assembled genome size	2.08Gb
Total length of contigs	2,085,722,329bp
Total number of contigs	572
Contig N50	76,960,141bp
Total length of scaffolds	2,085,726,029bp
Scaffolds N50	83,048,840bp
GC content	39.69%
Complete BUSCO	94.6%
Annotation features	Size or number
Number of protein-coding genes	38,178
Long terminal repeat (LTR) density	61.7%
Total repetitive sequence	1,836,775,398bp
Rate of repetitive sequence	88.06%

### Phylogenetic evolution and whole-genome duplication

**Figure 3:**
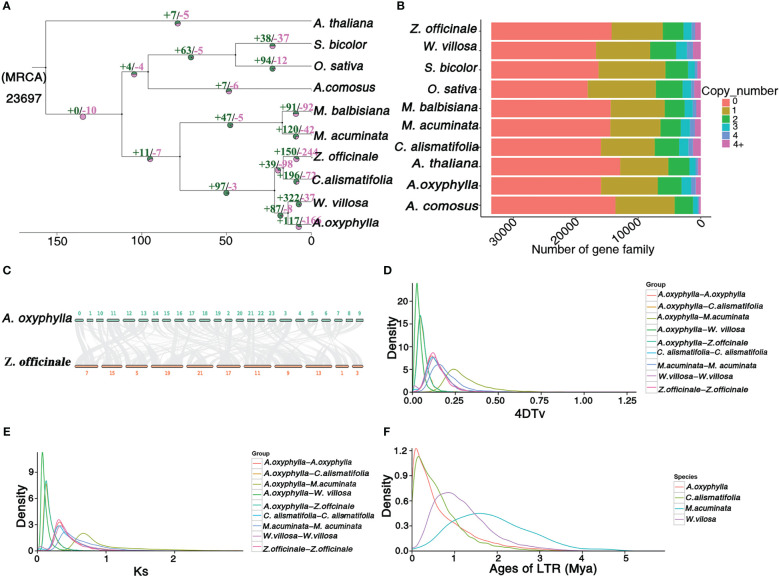
Genome and gene family evolution of *A. oxyphylla*. **(A)** Phylogenetic tree of 10 species *A. thaliana*, S. bicolor, O.sativa, A. comosus, M. balbisiana, M. acuminata, Z. officinale, C. alismatifolia, W. villosa, and *A. oxyphylla*. Gene family expansions/contractions are indicated in green/pink. Inferred divergence times (MYA, million years ago) are denoted at each node. **(B)** Gene categories used from all the species (the resource of this figure is from **Supplementary Data 1**). **(C)** Syntenic blocks between *A. oxyphylla* and Z. officinale. **(D, E)** Distribution of 4DTV values and Ks values of syntenic orthologous genes in the genomes of five species (M. acuminata, Z. officinale, C. alismatifolia, W. villosa, and *A. oxyphylla*. **(F)** Insertion time of LTRs in M. acuminata, C. alismatifolia, W. villosa, and *A. oxyphylla*.

In order to investigate the evolutionary status of *A. oxyphylla*, we conducted a comparative analysis of the available genomes of nine angiosperm species (**Figure 3A**). All species analyzed shared a total of 33,536 gene families, with 6,132 gene families being common across all species, while 440 gene families (equivalent to 1,747 genes) were unique to *A. oxyphylla* (**Supplementary Figure 6**). Using orthologs alignment of 832 single-copy gene families acquired in *A. oxyphylla* and nine other species, we constructed a phylogenetic tree (**Figures 3A, B**). The findings were consistent with the current understanding of the relationships among the ten species (Li et al., 2021a; Liao et al., 2022; Yang et al., 2022). This indicated that *A. oxyphylla* was firstly grouped with *W. villosa*, and these two genera were considered as a sister monophyletic group (Li et al., 2020a). *Z. officinale* and *C. alismatifolia* were the closest relatives, forming a parallel group that belonged to Zingiberaceae. The split time of *A. oxyphylla* and *W. villosa* was estimated at 13.7 (2.6-23) million years ago (Mya), while that of *Z. officinale* and *C. alismatifolia* was approximately 16.6 (2.6-23) Mya. The division of these two groups from Zingiberaceae occurred approximately 21.7 (2.6-23) Mya. In addition, based on the known divergence times of eudicots, monocots, Bromeliaceae, and Gramineae, we estimated that Zingiberaceae separated from Musaceae around 77 Mya (Li et al., 2021a), as shown in **Supplementary Figure 7**.

A total of 8,628 collinearity gene pairs were identified (**Figure 1B**) in our intergenomic analysis, which revealed strong linear relationships among these species of Zingiberaceae, and most of the chromosomes corresponded one to one. For example, 39,400 collinear genes between *A. oxyphylla* and *Z. officinale* were identified, indicating that 51.66% of the *A. oxyphylla* genome is colinear with the *Z. officinale* genome (**Figure 3C**).

This study aimed to estimate potential whole-genome duplication (WGD) events in the evolutionary history of *A. oxyphylla* by characterizing the distributions of four-fold synonymous third-codon transversion (4DTv) and synonymous substitution rates (Ks) of inter- and intra-*A. oxyphylla* and *Z. officinale*, *C. alismatifolia*, *W. villosa*, and *M. acuminate*. The sharp peak of Ks was about 0.05, and 4DTv was 0.125, in intra-*A. oxyphylla* and *C. alismatifolia*, *W. villosa*, and *Z. officinale*, suggesting their WGD events occurred after the divergence of Musaceae and Zingiberaceae (Ks,~0.75; 4DTv, ~0.25). We also determined that differentiation between *A. oxyphylla* and *W. villosa*. occurred approximately 14(9-19) Mya (Ks,~0.01;4DTv, ~0.025), while speciation of *A. oxyphylla*, *C. alismatifolia*, and *Z. officinale* occurred approximately 22(15-29) Mya (Ks,~0.02;4DTv, ~0.05) (**Figures 3D, F**). Additionally, we inferred the age of LTRs in four Musales species, finding that *A. oxyphylla* was the first to finish the expansion of LTRs (~0.01 Mya), followed by *C. alismatifolia* (~0.02 Mya), while *W. villosa* and *M. acuminate* expansions occurred much later (~1.00 and 1.50 Mya, respectively) (**Figure 3E**). In conclusion, the above collinearity analysis of inter- and intra-*A. oxyphylla* genomes helps to confirm the WGD event and LTR amplification involvement in *A. oxyphylla* speciation.

### Gene family analysis

**Figure 4:**
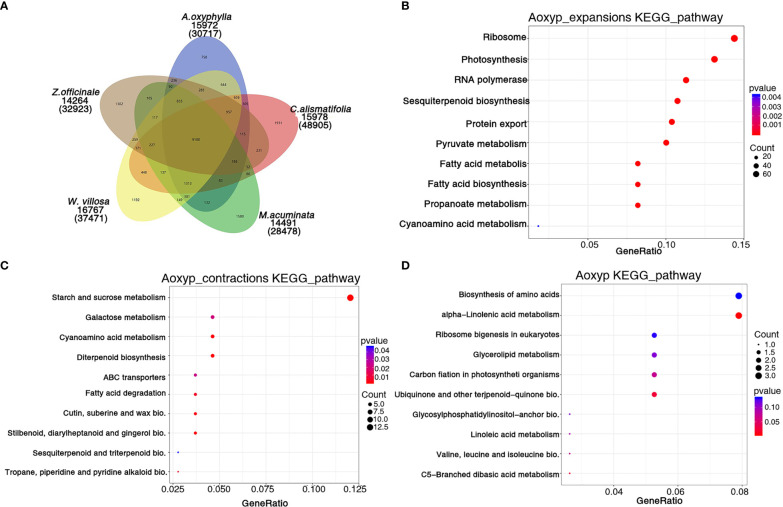
Gene family and functional enrichment analysis of *A. oxyphylla*. **(A)** The shared and unique gene families among five species (*M. acuminata, Z. officinale, C. alismatifolia, W. villosa* and *A. oxyphylla*). **(B–D)** KEGG enrichment analysis of expanded gene families, contracted gene families, and positively selected gene families in *A. oxyphylla*.

Based on the analysis of protein sequences from above 10 species, 30,717 genes were assigned to 15,972 families in *A. oxyphylla*, and 440 gene families, including 1,747 genes, were unique to *A. oxyphylla*, as illustrated in **Figure 4A**, **Supplementary Figure 6**, and **Supplementary Table 19**. To identify the shared and unique gene families, four other Musales species were selected for further analysis. The results indicated that 9,100 gene families were shared among the five species, and 758 unique gene clusters were identified in the *A. oxyphylla* genome (**Figure 4A**). Additionally, the specific KEGG pathway of *A. oxyphylla* genome was analyzed, and it was found that certain pathways, such as protein export, RNA transport, glutathione metabolism, and vitamin B6 metabolism, were significantly enriched (P<0.05) (**Supplementary Figure 8**).

After the divergence of *A. oxyphylla*, 117 gene families experienced significant expansion, while another 166 gene families showed significant contraction, as shown in **Figure 3A**. Our KEGG enrichment analysis indicates that the expanded gene families were involved in biosynthesis pathways such as ribosome, photosynthesis, sesquiterpenoid and triterpenoid biosynthesis, and pyruvate metabolism, relative to the biosynthesis of terpenoids in plastid (**Figure 4B**). In contrast, the contracted gene families showed enrichment in biosynthesis pathways including starch and sucrose metabolism, cyanoamino acid metabolism, diterpenoid biosynthesis, and stilbenoid, diarylheptanoid, and gingerol biosynthesis (**Figure 4C**). Additionally, our GO analysis revealed that the expanded gene families were mainly enriched in the malonyl-CoA biosynthetic process, acetyl-CoA carboxylase activity, and plastid, as shown in **Supplementary Figure 9B**. Conversely, the contracted gene families were mainly enriched in the defense response, extracellular region, and ADP binding (**Supplementary Figure 9C**).

When genes undergo strong positive selection, they play a critical role in generating novel functions within a species. In this study, we analyzed the genes that were subject to positive selection in *A. oxyphylla*. We identified a total of 106 positively selected genes and further performed KEGG analysis to explore their functions. This analysis revealed that several KEGG pathways, such as alpha-linolenic acid metabolism, ubiquinone, and other terpenoid–quinone biosynthesis, were significantly enriched (**Figure 4D**). GO analysis demonstrated that these positively selected genes were mainly associated with chloroplast organization, chloroplast structure, peptidyl-prolyl cis-trans isomerase activity, and serine-type endopeptidase activity (**Supplementary Figure 9D**). These positively selected genes, which are specific and expanded, could contribute to the biosynthesis of various secondary metabolites such as volatile terpenoids and flavones.

### Differentially expressed genes and characteristic metabolites analyses of *A. oxyphylla* from four different regions

Reverse-phase high-performance liquid chromatography (RP-HPLC) was utilized to analyze the levels of nootkatone, kaempferol, tectochrysin, and six other characteristic metabolites present in *A. oxyphylla* populations from 17 different regions (see **Supplementary Table 1**). The results of the variance analysis indicated that there were notable differences in four of these regions: Danzhou (A1-A3) and Baoting (A4-A6) received high comprehensive evaluation, while Nanning (B1-B3) and Zhangpu (B4-B6) received low comprehensive evaluation (**Supplementary Table 3-6**). Consequently, transcriptome and metabolome analyses of the same batch of materials from these four regions were performed with the aim of elucidating significant differences in the genes and metabolites.

A total of 103.49Gb of clean data, with 7.94 9.37 Gb per sample, were collected. On average, 86.32% of reads were mapped to the genome (**Supplementary Table 20**), resulting in the identification of 3,309 non-communal genes among the four regions (**Figure 2A**). The KEGG enrichment analysis revealed that 10 pathways, including phenylpropanoid biosynthesis, flavonoid biosynthesis, and stilbenoid, diarylheptanoid, and gingerol biosynthesis, were significantly enriched between A1-A6 and B1-B6 (**Figure 2B**). Additionally, GO analysis highlighted significant enrichment in photosystem II, protein polymerization, transferase activity transferring acyl groups other than amino-acyl groups, and terpene synthase activity (**Supplementary Data 6**). A total of 302 common metabolites were found among the four regions (**Figure 2D**), which were mainly enriched in ubiquinone and other terpenoid–quinone biosynthesis, fatty acid biosynthesis, and flavone and flavonol biosynthesis pathways (**Figure 2E**). These pathways allow for insight into the metabolic processes underlying the significant variations in metabolites content among the different regions of *A. oxyphylla*.

In order to unravel the molecular mechanism underlying the variation in *A. oxyphylla* quality across the different regions, we performed an integrated analysis of the transcriptome and metabolome to identify the top 30 hub genes central to the biosynthesis of nootkatone and kaempferol, which are the major metabolites in *A. oxyphylla* fruit. Using the fragments per kilobase of exon model per million mapped fragments (FPKM) data, we created a heatmap visualization to map the distribution of these 30 genes across different regions (**Figures 2C, F**). Our results showed that the genes involved in nootkatone biosynthesis were highly expressed in the A1-A6 regions, which have higher pharmacodynamic components (**Figure 2C**). All the annotated genes were functional except for asm_4.69, which was predicted to be an ethylene-responsive transcription factor 3, suggesting its critical regulatory function. Isopentenyl diphosphate delta-isomerase I (IPPI) (asm_23.1717) is the key enzyme of terpenoid synthesis, and it was highly expressed in all regions, representing its essential roles in sesquiterpene biosynthesis. Among the top 30 ranked genes involved in kaempferol biosynthesis, five transcription factors (TFs) (asm_13.1044, 23.348, 18.685, 12.1808, and 4.757) were identified, including MYB98 (MYB98, KAN4, DIVARICATA), KAN4, DIVARICATA, and two bZIP TFs, implying their potential roles in flavonol biosynthesis (**Figure 2F**).

**Figure 5:**
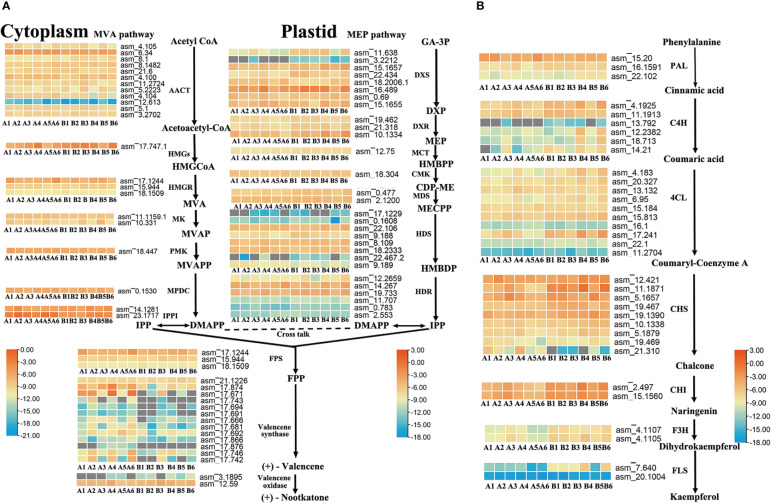
Terpenoid and flavonoid metabolic pathways involved in the biosynthesis of nootkatone and kaempferol in *A*. *oxyphylla*. Heatmap showing the expression level of candidate genes involved in nootkatone **(A)** and kaempferol **(B)** synthesis in different regions. Based on the million mapped fragments (FPKM) value, genes with the identity>70% were selected on the nootkatone and kaempferol biosynthesis pathway; for functional annotation of related genes, see **Supplementary Data 4, 5**. The FPKM values are log2-based. Red and blue indicate high and low expression levels, respectively. Enzyme abbreviations: MVA pathway: AACT (acetyl-CoA acetyltransferase); HMGS (3-hydroxyl-3-methylglutaryl-CoA synthase); HMGR (3-hydroxy-3-methylglutaryl-CoA reductase); MK (mevalonate kinase); PMK (phosphomevalonate kinase); MPDC (pyrophosphomevalonate diphosphate decarboxylase); IPPI (IDP isomerase); MEP pathway: DXS (1-deoxy-D-xylulose-5-phosphate synthase); DXR (1-deoxy-D-xylulose-5-phosphate reductoisomerase); MCT (2-C-methyl-D-erythritol-4-phosphate cytidylyltransferase); CMK (4-(cytidine 5-diphospho)-2-C-methylerythritol kinase); MDS (2-C-methul-D-erytyritol 2,4-cyclodiphosphate synthase); HDS (4-hydroxy-3-methylbut-2-enyl-diphosphate synthase); HDR (4-hydroxy-3-methylbut-2-enyl-diphosphate reductase); PAL (phenylalanine ammonia-lyase); C4H (cinnamate-4-hydroxylase); 4CL (4-coumarate CoA ligase); CHS (chalcone synthase); CHI (chalcone isomerase); F3H (flavanone 3-hydroxylase); FLS (flavonol synthase). Compound abbreviations: HMGCoA (3-hydroxyl-3-methyglutaryl-CoA); MVA (mevalonate); MVAP (mevalonate-5-phosphate); MVAPP (mevalonate-5-diphosphate); IPP (isopentenyl diphosphate); DMAPP (dimethylallyl diphosphate); GA-3P (D-Glyceraldehyde 3-phosphate); DXP (1-Deoxy-D-xylulose 5-phosphate); MEP (2-C-Methyl-D-erythritol 4-phosphate); HMBPP (4-(Cytidine 5’-diphospho)-2-C-methyl-D-erythritol); CDP-ME (2-Phospho-4-(Cytidine 5’-diphospho)-2-C-methyl-D-erythritol); MECPP (2-C-Methyl-D-erythritol 2,4-cyclodiphosphate); HMBDP (4-Hydroxy-3-methylbut-2-enyl-diphosphate); FPP (farnesyl diphosphate).

To further identify the key enzyme genes responsible for the differences in nootkatone and kaempferol content across the four regions of *A. oxyphylla*, we compared and screened the genes involved in terpenoid and flavonols backbone biosynthesis. Our results identified 87 and 35 genes, respectively, in relation to their relevant biosynthesis pathways. The enzymes AACT, DXS, HDS, HDR, and valencene synthase showed higher copy numbers in the nootkatone biosynthesis pathway, while the PAL, C4H, 4CL, and CHS genes exhibited higher copy numbers in the kaempferol biosynthesis pathway (**Figure 5**), potentially indicating rate-limiting enzymes. However, on examining their expression profiles in the four regions, few genes in the terpenoid and flavonoid backbone biosynthesis pathway were specifically highly expressed, except valencene synthase, which belongs to the downstream genes in the volatile terpenoid biosynthesis pathway. Therefore, we postulate that terpene synthases (TPSs) are likely responsible for the region-specific differences in sesquiterpenoids accumulation observed in *A. oxyphylla* fruit.

### Genome-wide detection of TPS genes in *A. oxyphylla*

**Figure 6:**
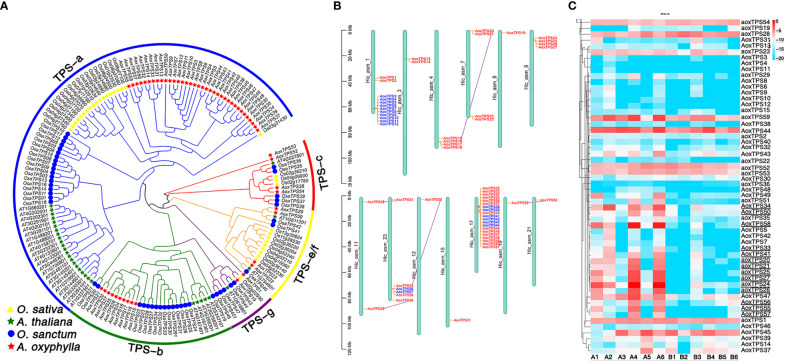
Analysis of TPS gene family in *A*. *oxyphylla*. **(A)** Phylogenetic tree of TPS genes from *A. oxyphylla* (59 genes), *O. sativa* (32 genes), *A. thaliana* (31 genes), and *O. sanctum* (47 genes). The outer circle and branch colors represent different TPS gene subfamilies. **(B)** Schematic map presentation of the genomic localization of 59 *AoxTPSs*. Blue represents tandem duplicates, and purple-linked genes represent genome-wide replication. **(C)** Heatmap (row scale) showing the differentially expressed of *AoxTPSs* according to the transcriptome data in four regions. Uppercase AOX represents *AoxTPSs* from a or b subfamily, and lowercase aox represents *AoxTPSs* from other subfamilies. Underlined *AoxTPSs* represents highly expressed in A1-A6 areas. Sample class: H1 (A1-A3): Danzhou; H2(A4-A6): Baoting; L1 (B1-B3): Nanning; L2(B4-B6): Zhangpu.

TPSs play a crucial role in catalyzing GPP, FPP, and GGPP, which produce the skeletons of monoterpenes, sesquiterpenes, and diterpenes, respectively. These enzymes have evolved different-sized subfamilies in various plant species, but typical plant TPSs are a valuable tool to examine plant evolution since they belong to a mid-sized gene family that is conserved more by lineage than by function (Jia et al., 2022). The previous phylogenetic tree of TPS genes from gymnosperms and angiosperms divided the TPSs into seven subfamilies (TPS-a to TPS-g) (Bohlmann J and Croteau, 1998; Dudareva et al., 2003; Martin and Bohlmann, 2004). Among them, TPS-d is specific to gymnosperm (Chen et al., 2011). In this study, we identified 56 putative *AoxTPSs* based on the assembly genome of *A. oxyphylla* and performed phylogenetic analysis to understand their evolutionary relationship with *O.sativa*, *A. thaliana*, and *O. sanctum* (**Figure 6A**). We observed that 165 TPSs were classified into five subfamilies: TPS-a, TPS-b, TPS-c, TPS-e/f, and TPS-g. Most of the predicted *AoxTPS* genes (46) were clustered into TPS-a (36) and TPS-b (10) subfamilies, suggesting their significant expansion in the genome of *A. oxyphylla* and their possible contribution to mass-producing sesquiterpenoids and monoterpenoids in the fruit. The TPS-c, TPS-e/f, and TPS-g subfamilies in the phylogenetic tree showed 3, 4, and 1 members of *AoxTPSs*, respectively, and the TPS-b and TPS-g subfamilies were mainly responsible for producing monoterpenoids; thus, the TPS-b and TPS-g subfamilies were shown in a combined state with the representation of separate clusters. *AoxTPS* 1, *AoxTPS* 52, and *AoxTPS*53 were not attributed to any subfamilies, indicating that these gene copies originated from dispersed or segmental duplication after species divergence (Li et al., 2022). These 59 *AoxTPSs* were distributed on 13 chromosomes, with 20 genes (*AoxTPS*32-*AoxTPS* 51) located on the chromosome 17 (**Figure 6B**). Terpenoid biosynthesis genes are generally organized into tandem metabolic gene clusters (Osbourn, 2010; Nützmann and Osbourn, 2014). We found three tandem gene clusters distributed on chromosome 1, 23, and 17, with 10, 2, and 6 *AoxTPS* genes located, respectively, suggesting that tandem duplication events have participated in the expansions of *AoxTPSs*. Further validation is needed to establish whether these expansions contribute to its terpene biosynthesis. We also compared the expression profiles of *AoxTPSs* in four different regions (**Figure 6C**) and found 14 *AoxTPSs* (consisting of eight genes belonging to the TPS-a subfamily and six genes belonging to the TPS-b subfamily) that exhibited higher transcript abundance in high pharmacodynamic components regions (A1-A6). Some key genes were further validated by qRT-PCR, and the expression level of valencene synthase gene copy *AoxTPS* 34 and *AoxTPS* 50 was consistent with the accumulation of nootkatone from 17 different regions (**Supplementary Figure 10**). These results above suggest that these genes are important in the different quality formation of *A. oxyphylla* among the 17 regions based on nootkatone content.

## Discussion

*A. oxyphylla* is one of the “Four Famous South Medicines” in China, which significantly contributes to the understory planting economy. The genome, metabolome, and transcriptome data collected for *A. oxyphylla* constitute essential genetic, genomic, and transcriptome resources that can be utilized in future research to comprehend its evolution, biosynthesis of pharmacodynamics components, and quality difference formation. These resources hold significance for studying other species of the Zingiberaceae family and have economic, ecological, and research value. The basic number and ploidy level of Zingiberaceous species are various. In this study, we report the somatic chromosome number of *A. oxyphylla* as 2n=48, which agrees with the previous cytological study by Saenprom et al. (2018). However, our cytomorphological and genome survey results indicate that, contrary to previous beliefs (Eksomtramage and Boontum, 1995; Saenprom et al., 2018), *A. oxyphylla* is a diploid rather than tetraploid as it belongs to the Alpinia genus. Our analysis involved a combination of PacBio and Hi-C technology, which resulted in the assembly of a 2.08 Gb chromosome-scale genome with a contig N50 of 76.96 Mb and scaffold N50 of 83.04 Mb. Moreover, 96.08% of contigs were anchored to the 24 chromosomes. The quality of this assembly is superior to that of recently published species from the Zingiberaceae family, including *W. villosa* (contig N50 of 9.13 Mb) (Yang et al., 2022), *Z. officinale* (contig N50 of 12.68 Mb) (Cheng et al., 2021b), *A. tsao-ko* (contig N50 of 4.8 Mb) (Li et al., 2022), and *C. alismatifolia* (N50 of 57.51Mb) (Liao et al., 2022). Additionally, we annotated 38,178 genes, which is more than *Z. officinale* (36,503) but less than *C. alismatifolia* (57,534) (Liao et al., 2022) and *W. villosa* (42,588) (Yang et al., 2022). The divergence between *A. oxyphylla* and *W. villosa* was approximately 13.7 Mya, while the speciation of *C. alismatifolia* and *Z. officinale* occurred around 16.9 Mya, which is earlier than the estimation of Liao et al. (2022), who proposed that *C. alismatifolia* and *Z. officinale* diverged approximately 11.9 Mya. The distributions of Ks and substitution rate of 4DTv suggest that a recent WGD event was shared by *A. oxyphylla*, *C. alismatifolia*, *W. villosa*, and *Z. officinale*. This observation corroborates the recent WGD reported in other species (Li et al., 2021a; Liao et al., 2022; Yang et al., 2022) and provides further evidence of the shared WGD in the Zingiberaceae family (Cheng et al., 2021b).

The main pharmacodynamic components of FAO are terpenoids and flavonoids, with sesquiterpene nootkatone and flavonal kaempferol having the highest content, respectively. Nootkatone is predominantly found in the seeds, which is the traditional medicinal part, while kaempferol is mainly deposited in the capsules (Chen et al., 2014). Compared to other Zingiberaceae species such as *W. villosa*, *A. oxyphylla* has experienced significant expansion in 117 gene families. KEGG terms related to pyruvate metabolism and sesquiterpenoid and triterpenoid biosynthesis were significantly enriched in these gene families, and GO analysis showed that expanded gene families were mainly enriched in the malonyl-CoA biosynthetic process, acetyl-CoA carboxylase activity, and plastid. This suggests that *A. oxyphylla* has accumulated genes involved in terpenoid and flavonoids synthesis in recent evolutionary history.

Geo-herbalism is an important index reflecting the quality of traditional Chinese medicine as it is mainly influenced by heredity factors and the environment. One crucial element in geo-herbalism is the content of medicinal ingredients. Accordingly, this study analyzed the gene expression differences in the nootkatone and kaempferol biosynthesis pathway among samples from four different *A. oxyphylla*-growing regions. The results indicated that ethylene-responsive transcription factor IPPI exhibited a critical regulation function in sesquiterpene biosynthesis. Ethylene-responsive transcription factors also play an important role in various abiotic stresses, and they can induce terpenoid synthesis (*OsTPS33, OsTPS14, OsTPS3*) in *O. sativa* in a drought stress environment (Jung et al., 2021) and accelerate the metabolic flux of tanshinone (a type of diterpene) accumulation in *S. miltiorrhiza* (Bai et al., 2018). The interconversion of isopentenyl diphosphate (IPP) and dimethylallyl diphosphate (DMAPP) is mediated by IPPI, which is the only enzyme shared by the mevalonic acid (MVA) and methylerythritol phosphate (MEP) pathways. Many plants contain two IPPI isoforms with different expression profiles encoding proteins and subcellular locations. It has been reported that OsIPPI1 is predominantly responsible for the synthesis of MVA pathway-derived terpenoids, while OsIPPI2 is responsible for the synthesis of MEP pathway-derived terpenoids, such as chlorophylls and carotenoids (Jin et al., 2020). Like many other secondary metabolites, flavonoids play important roles in the interaction of plants with their environment. Additionally, three MYB (MYB98, KAN4, DIVARICATA) and two bZIP TFs were found to be closely related to flavonols biosynthesis in *A. oxyphylla*. For example, KAN4, which belongs to the MYB family, has been previously shown to regulate the biosynthesis of flavonols in Arabidopsis seeds (Gao et al., 2010). Similarly, another MYB gene, *Sm*MYB98, can activate the transcription of the *SmGGPPS1, SmPAL1, and SmRAS1* genes and play a positive regulatory role in the synthesis of tanshinone in *S. miltiorrhiza* (Hao et al., 2020). bZIP was found to focus on the regulation of genes in the upstream synthesis of phenylalanine but inhibit the formation of flavones (flavonol synthase) (Mei et al., 2021). Hence, these genes or TFs may contribute to the biosynthesis of sesquiterpenoids and flavonols in *A. oxyphylla*.

The present study investigated the genes that contribute to the biosynthesis of kaempferol and nootkatone in *A. oxyphylla*. Specifically, the study focused on PAL, C4H, 4CL, CHS, AACT, DXS, HDS, HDR, and valencene synthase, which were found to have expanded gene families in *A. oxyphylla*. PAL catalyzes the first step of flavonoid biosynthesis pathway and was shown in a previously conducted study to be significantly upregulated as the *A. oxyphylla* fruit matures (Pan et al., 2022). Among these genes, C4H, 4CL, and CHS are involved in regulating other primary steps of flavonoid biosynthesis and show differential expression in different tissues of *A. oxyphylla* (Yuan et al., 2021). AACT, DXS, HDS, and HDR encode key enzymes of terpenoid backbone biosynthesis, and AACT is the initiation enzyme of the MVA pathway, which predominantly provides the precursors for the cytosolic biosynthesis of sesquiterpenoids and for terpenoid biosynthesis in mitochondria. DXS, HDS, and HDR serve as rate-limiting or regulatory enzymes in the MEP pathway and are preferably used for the biosynthesis of monoterpenoids, diterpenoids, carotenoids, and other compounds (Tholl, 2015). But the expression of all the aforementioned genes did not show any evident difference between the A and B regions, except valencene synthase, which belongs to the TPS family and showed considerable expansion in *A. oxyphylla*. This coincides with reports on other plant species, such as *O. sanctum* (Kumar et al., 2018), *W. villosa* (Yang et al., 2022), *Z. officinale* (Cheng et al., 2021b), and Citrus grandis ‘Tomentosa’ (Xian et al., 2022). Previous studies have suggested that the expansion of the TPS-a and TPS-b subfamilies contributes to the diversity and content enrichment of sesquiterpenoids and monoterpenoids, respectively (Chaw et al., 2019; Yang et al., 2022). In the current study, eight AoxTPS genes belonging to the TPS-a or TPS-b subfamilies (AoxTPS34, AoxTPS50, AoxTPS58, AoxTPS33, AoxTPS41, AoxTPS55, AoxTPS56, and AoxTPS57) were highly expressed in most regions with higher pharmacodynamic components. This finding suggests that these genes may play a role in the regionally different quality formation of *A. oxyphylla*. Furthermore, these genes were validated and selected as candidate genes for utilization in molecular breeding.

## Conclusion

This study presents a high-quality chromosome-level reference genome of *A. oxyphylla*. We conducted comprehensive genomic, transcriptomic, and metabolic analyses on population materials from four different plant regions. Our findings reveal that materials from the Hainan region contain higher levels of pharmacodynamic components and confirm that their geo-herbalism properties are attributed to the higher content of nootkatone. Furthermore, we identified the valencene synthase gene, which is likely responsible for the efficient nootkatone synthesis ability across different regions. Therefore, these results contribute significantly to the assessment of *A. oxyphylla* quality in production practices and also to functional genomic research and genome-assisted breeding.

## Data availability statement

The datasets presented in this study can be found in online repositories. The names of the repository/repositories and accession number(s) can be found in the article/**Supplementary material**.

## Author contributions

KP, SZ and JZ designed the project and contributed to the original concept of the manuscript. SZ performed *de novo* genome assembly and annotation and analyzed all the data. KP collected the material from 17 regions and wrote the manuscript. SD completed the karyotype analysis of *A. oxyphylla* and revised the manuscript. BG analyzed the transcriptome and metabolism data. JL performed the DNA/RNA extraction and RT-qPCR analysis. ML and SL participated in the RP-HPLC analysis. All authors contributed to the article and approved the submitted version.
