# Supplementary material for: Chromosome-level genome and multi-omics analyses provide insights into the geo-herbalism properties of Alpinia oxyphylla
Source: Front Plant Sci. 2023 Jun 8;14:1161257. doi: 10.3389/fpls.2023.1161257 (PMC10285302; doi:10.3389/fpls.2023.1161257)
Supplement: Supplementary file 2 [file Image_1.pdf]

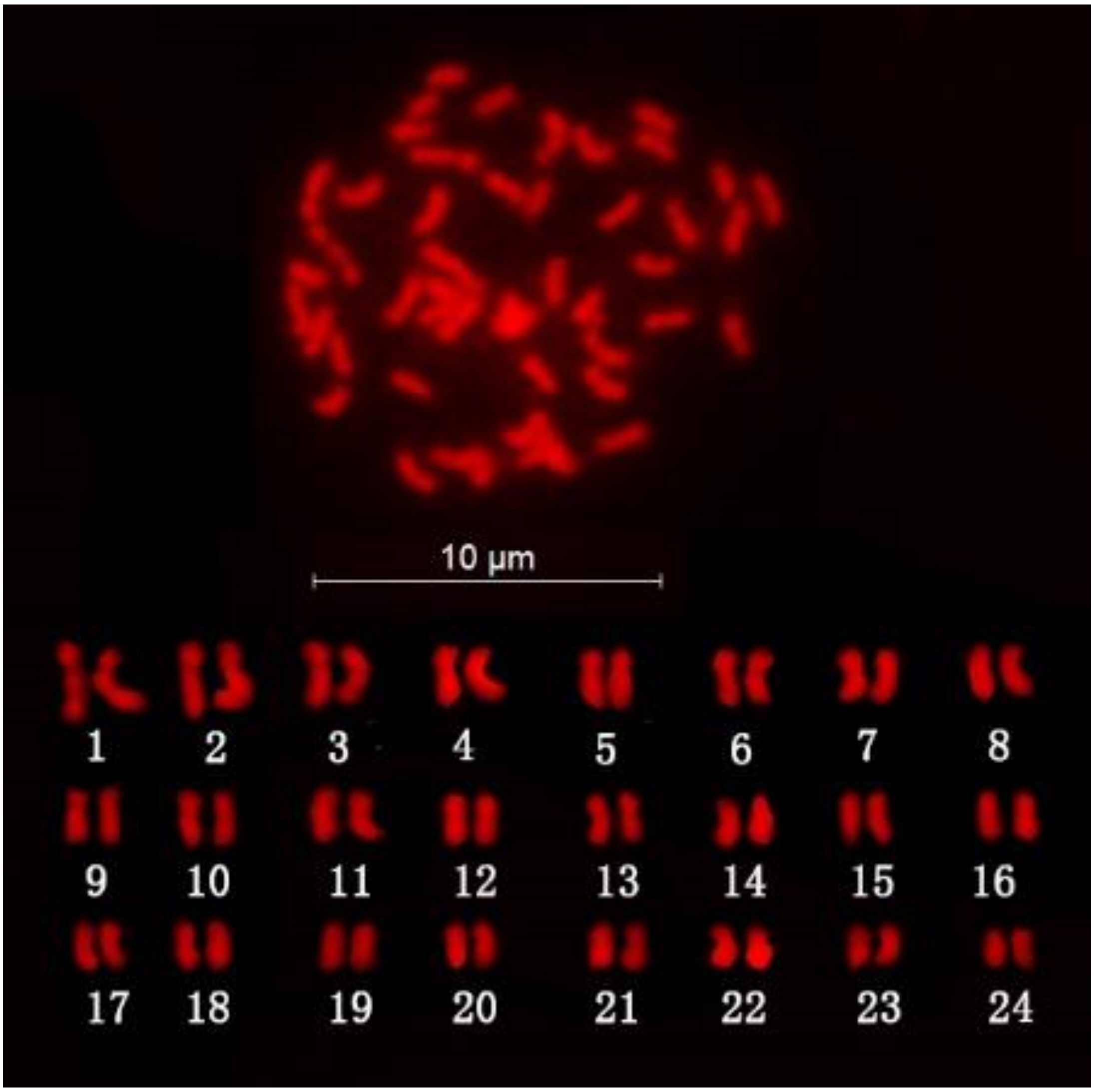

Supplementary Figure 1. Somatic metaphase plates and karyograms of *A. oxyphylla.*

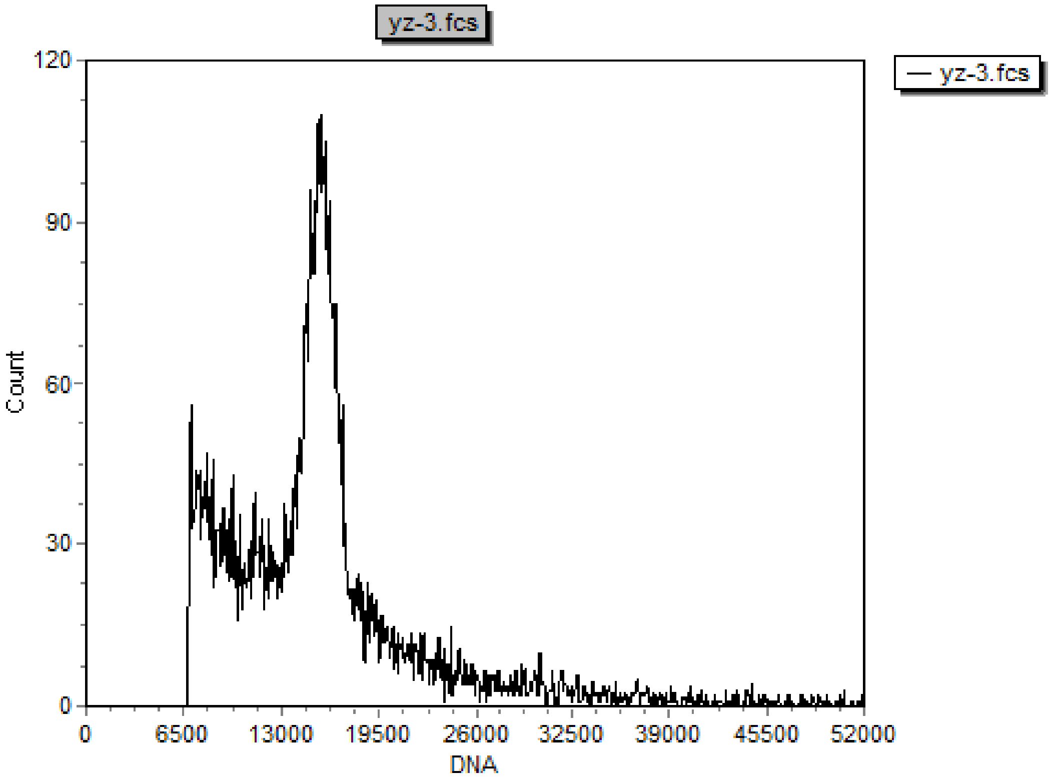

Supplementary Figure 2. The flow cytometry determination result of *A. oxyphylla.*

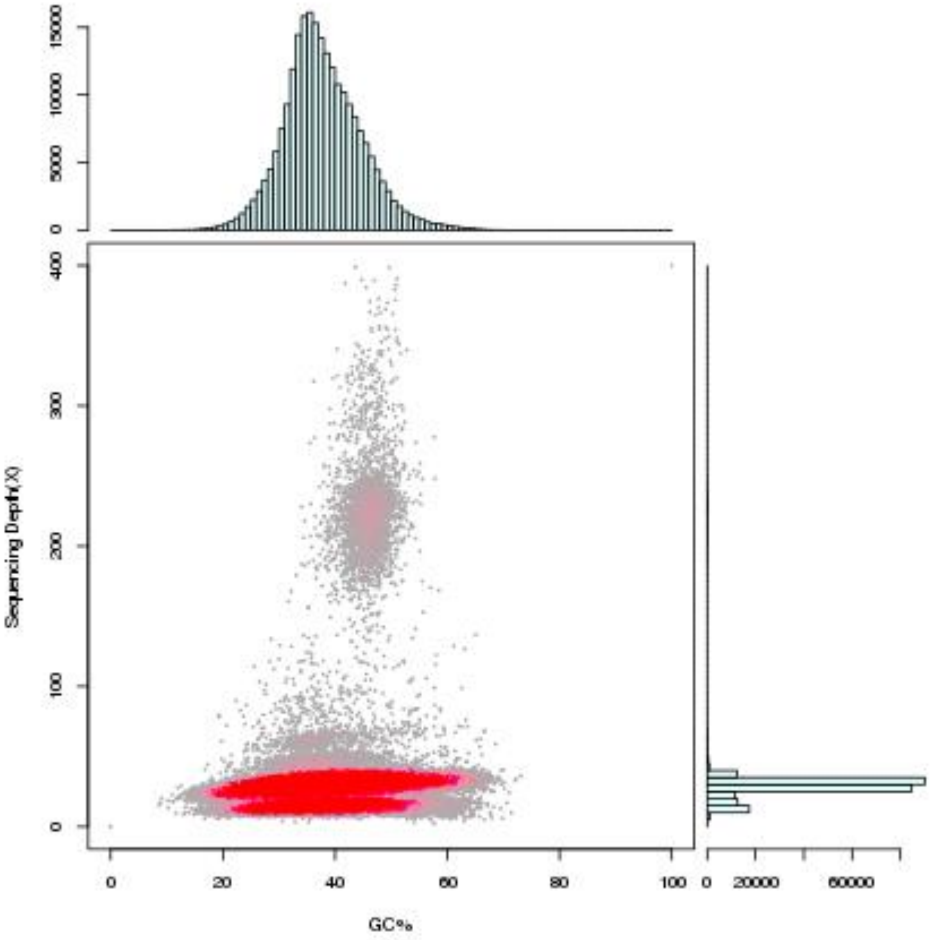

Supplementary Figure 3. The size estimation of the *A. oxyphylla* genome. The genome size was estimated by calculating the distribution of 19-mer frequency in the sequencing reads. The x-axis is the depth (X), the y-axis is the content of GC and proportion of sequences that represent the frequency at that depth divided by the total frequency of all depths.

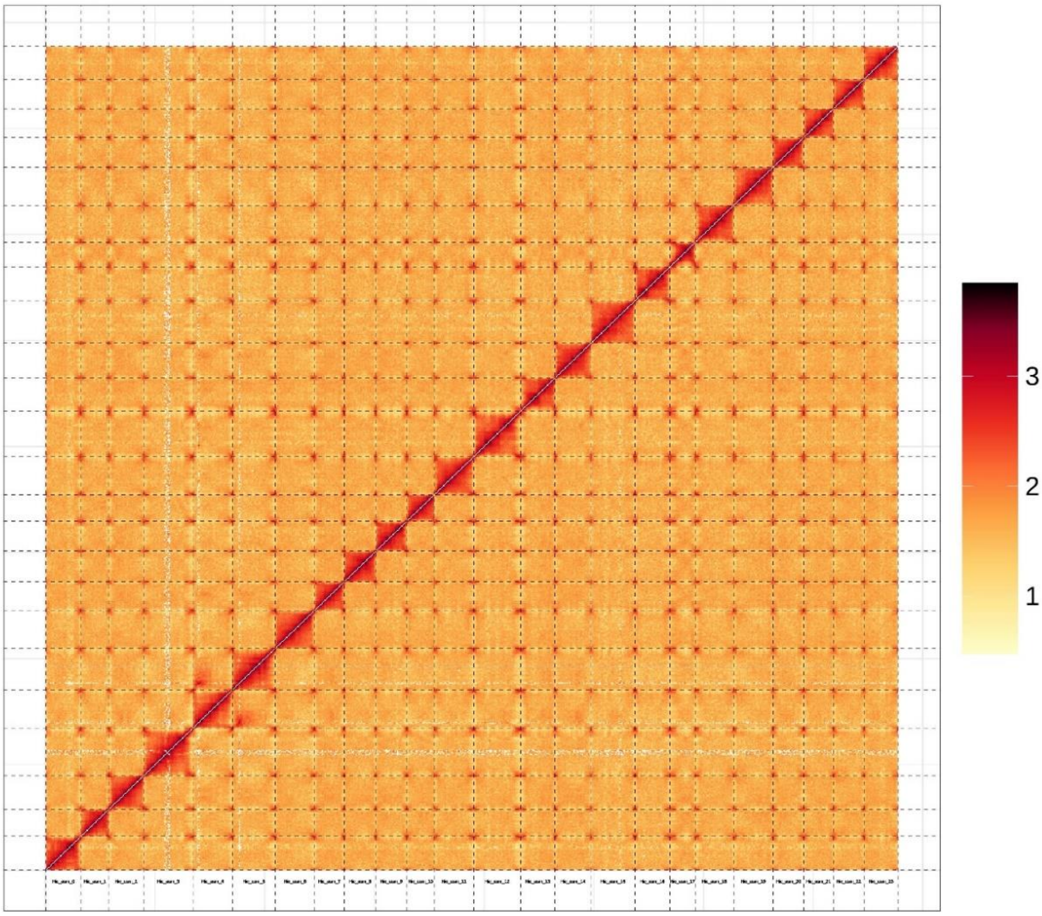

Supplementary Figure 4. The Hi-C chromation interaction map for the 24 pseudomolecules of *A. oxyphylla* genome

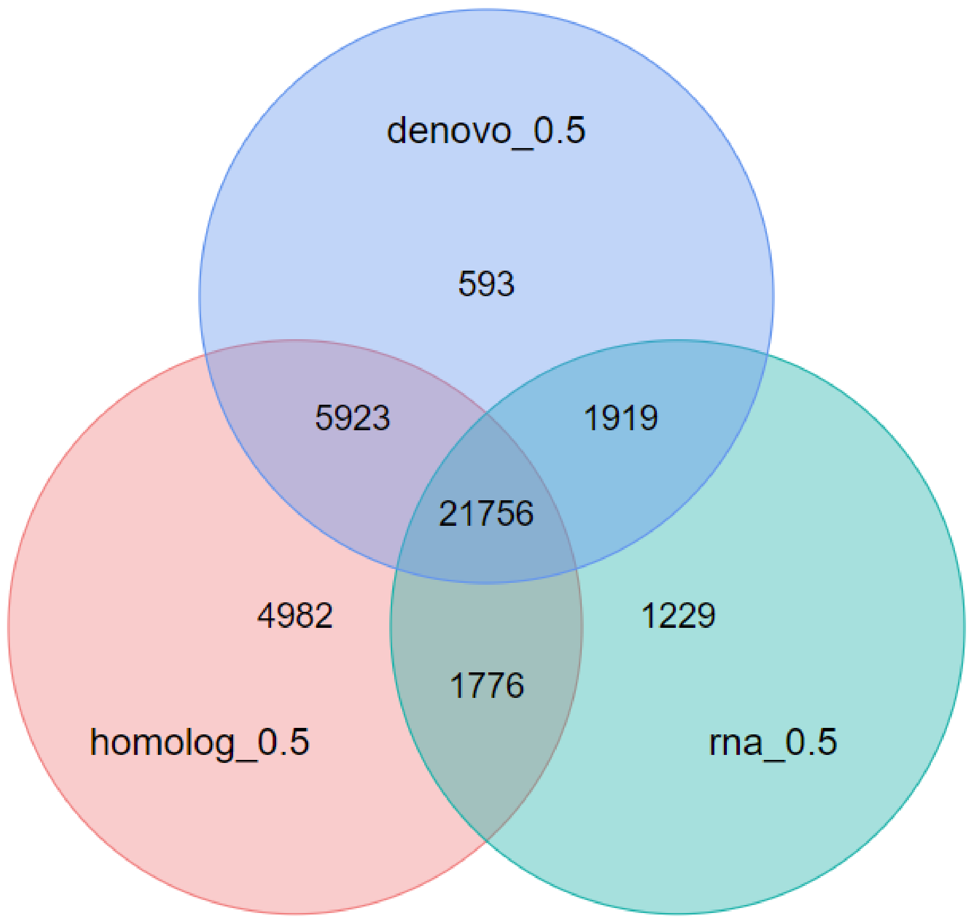

Supplementary Figure 5. Statistics of evidence support for gene sets.

Note :Denovo, EVM integrate the genes supported by Denovo prediction;Homolog, a gene supported by homologous prediction during EVM integration;RNA, genes supported by RNA-SeQ during EVM integration;Each evidence support is based on genetic overlap greater than 50%.The number represents the number of genes.

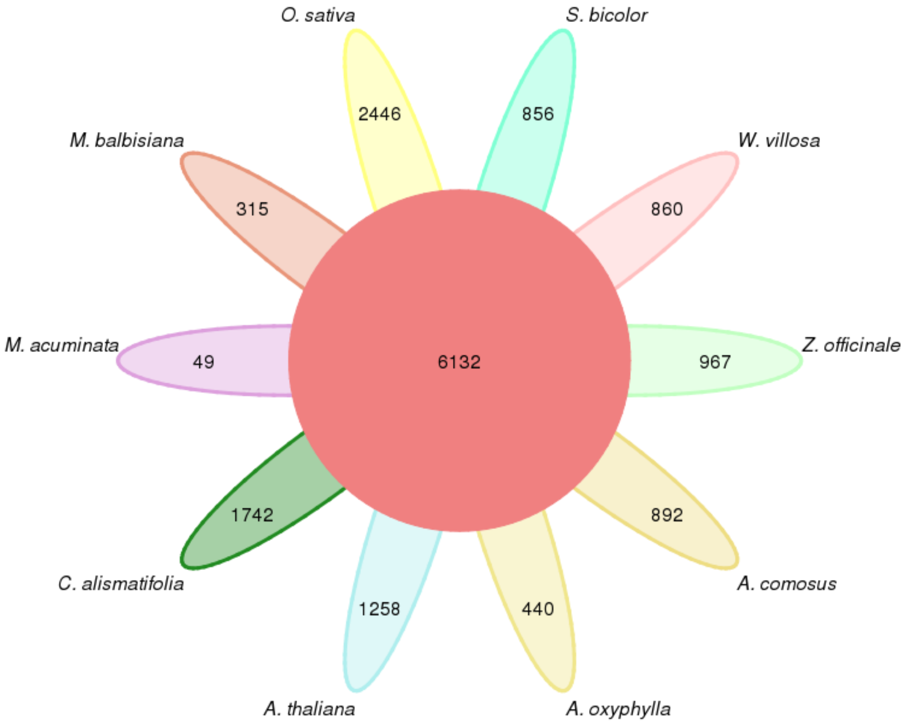

Supplementary Figure 6. Syntenic blocks between *A. oxyphylla* and other 9 species.

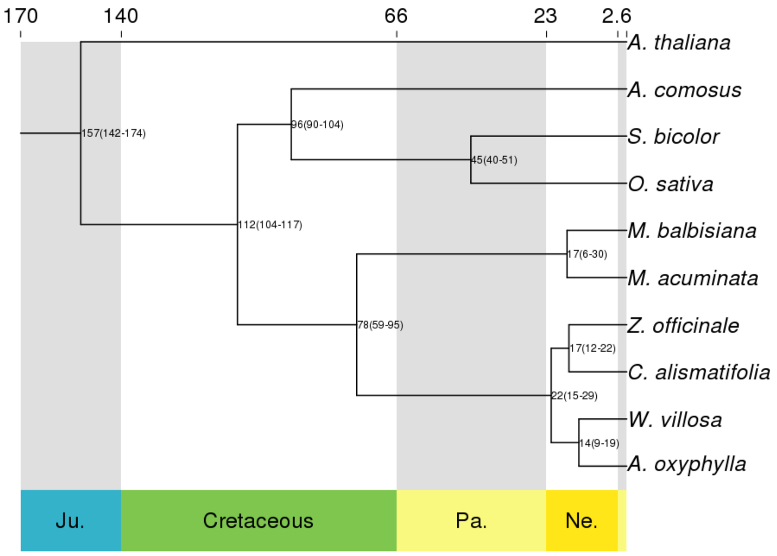

Supplementary Figure 7. Divergence times of *A. oxyphylla* and other 9 species.

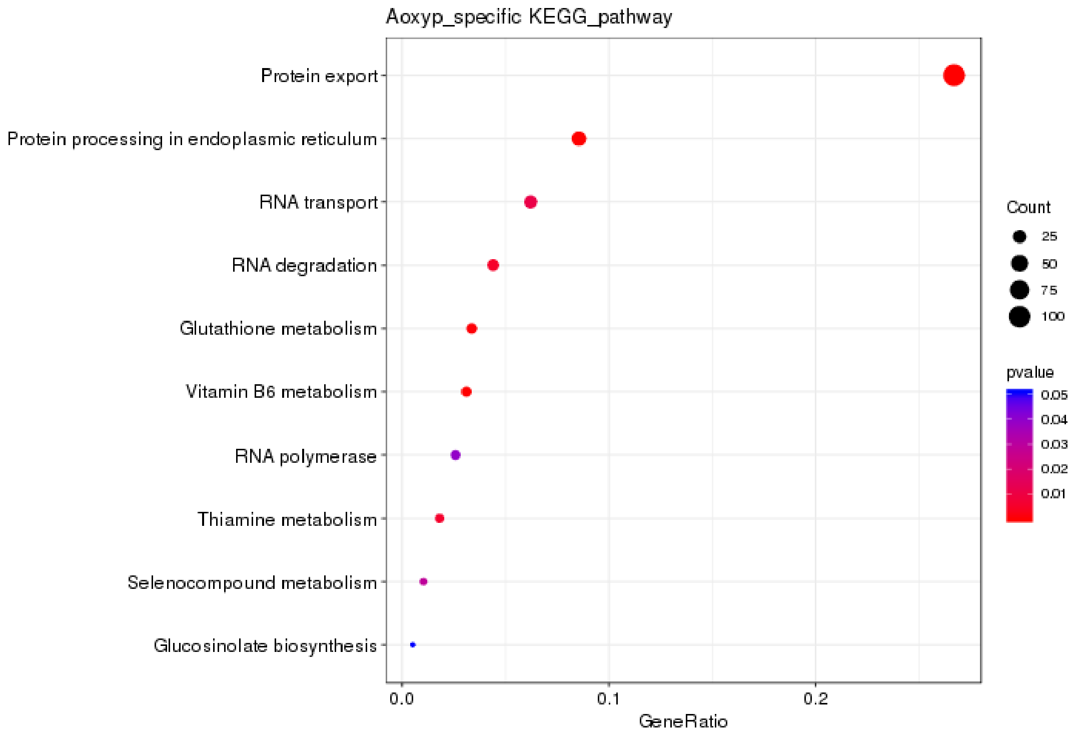

Supplementary Figure 8. KEGG pathway enrichment dotplot of *A. oxyphylla.*

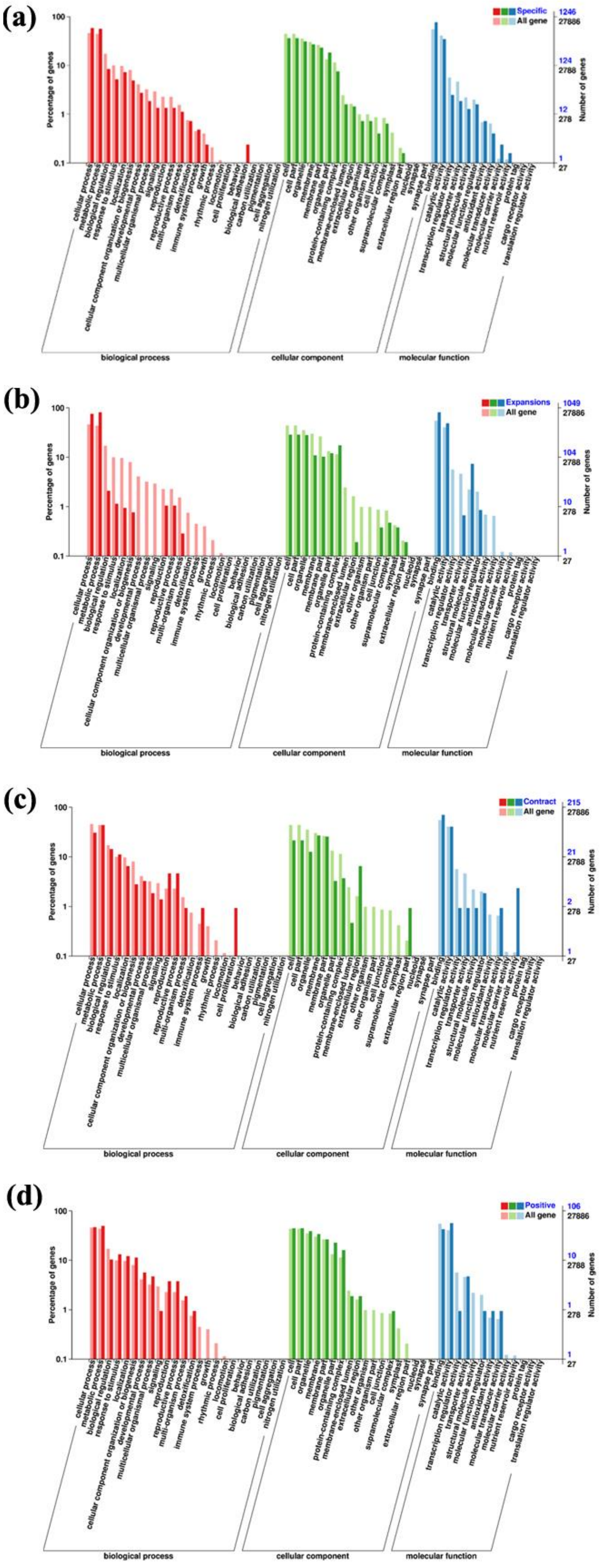

Supplementary Figure 9. GO enrichment analysis of specific gene families(a), expansion gene families(b), contraction gene families(c) and positive selected gene families(d) in *A. oxyphylla.*

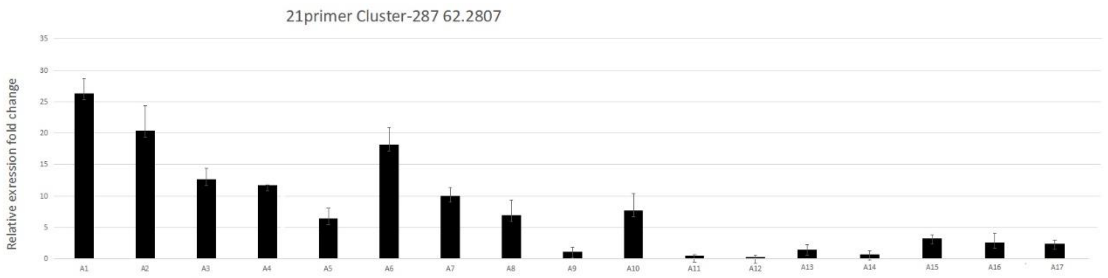

Supplementary Figure 10. qRT-PCR results of primer 21 (valencene synthase gene copy *AoxTPS* 34) on 17 different regions (A1-A17: Danzhou (DZ), Dongfang (DF), Baisha (BS), Qiongzhong (QZ), Wuzhishan (WZS), Baoting (BT), Wanning (WN), Haikou (HK), Fangchenggang (FCG), Nanning (NN), Rongxian (RX), Xinyi (XY), Gaozhou (GZ), Yangchun (YC), Xishaungbanna (XSBN), Zhangpu (ZP), Fuzhou (FZ).

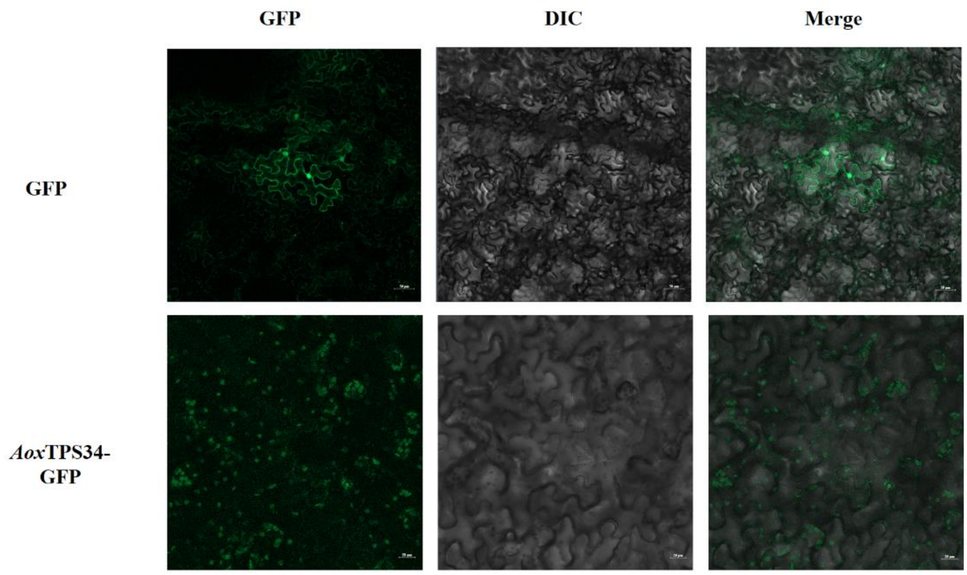

Supplementary Figure 11 Subcellular localization of TPS34-GFP fusion protein in tobacco leaves. The subcellular location of the fusion protein was observed by confocal microscopy, which proved that the subcellular location of the TPS34 gene is in the chloroplast
