# Supplementary material for: Chromosome-level genome and multi-omics analyses provide insights into the geo-herbalism properties of Alpinia oxyphylla
Source: Front Plant Sci. 2023 Jun 8;14:1161257. doi: 10.3389/fpls.2023.1161257 (PMC10285302; doi:10.3389/fpls.2023.1161257)
Supplement: Supplementary file 3 [file Table_1.pdf]

Supplementary Table 1. Gene categories used from all the species

**Name**	**Num**	**Copy_number**
A. comosus	19880	0
A. oxyphylla	17564	0
A. thaliana	20638	0
C. alismatifolia	17558	0
M. acuminata	19045	0
M. balbisiana	19112	0
O. sativa	15425	0
S. bicolor	17164	0
W. villosa	16769	0
Z. officinale	19272	0
A. comosus	9470	1
A. oxyphylla	9101	1
A. thaliana	7704	1
C. alismatifolia	8602	1
M. acuminata	8035	1
M. balbisiana	8648	1
O. sativa	10910	1
S. bicolor	10709	1
W. villosa	8662	1
Z. officinale	8180	1
A. comosus	2948	2
A. oxyphylla	3789	2
A. thaliana	3374	2
C. alismatifolia	3918	2
M. acuminata	3254	2
M. balbisiana	3183	2
O. sativa	4277	2
S. bicolor	3624	2
W. villosa	4168	2
Z. officinale	3315	2
A. comosus	761	3
A. oxyphylla	1551	3
A. thaliana	981	3
C. alismatifolia	1484	3
M. acuminata	1475	3
M. balbisiana	1301	3
O. sativa	1373	3
S. bicolor	1079	3
W. villosa	1718	3
Z. officinale	1292	3
A. comosus	223	4
A. oxyphylla	730	4

A. thaliana	399	4
C. alismatifolia	726	4
M. acuminata	770	4
M. balbisiana	574	4
O. sativa	507	4
S. bicolor	414	4
W. villosa	961	4
Z. officinale	579	4
A. comosus	254	4+
A. oxyphylla	801	4+
A. thaliana	440	4+
C. alismatifolia	1248	4+
M. acuminata	957	4+
M. balbisiana	718	4+
O. sativa	1044	4+
S. bicolor	546	4+
W. villosa	1258	4+
Z. officinale	898	4+

Supplementary Table 2. Karyotype analysis parameters of *A. oxyphylla*

N.O.	LS	LL	LT	CL	AR	CT	CLT
1	2.12	4.11	6.23	1.48	1.94	sm	L
2	2.21	3.97	6.18	1.47	1.8	sm	L
3	2.18	3.02	5.2	1.24	1.39	m	M2
4	2.09	3.02	5.11	1.22	1.44	m	M2
5	1.84	2.80	4.64	1.10	1.52	m	M2
6	1.92	2.61	4.53	1.08	1.36	m	M2
7	1.89	2.42	4.31	1.03	1.28	m	M2
8	1.82	2.39	4.21	1	1.31	m	M1
9	1.78	2.34	4.12	0.98	1.31	m	M1
10	1.78	2.35	4.12	0.98	1.31	m	M1
11	1.71	2.35	4.06	0.96	1.37	m	M1
12	1.70	2.27	3.97	0.94	1.33	m	M1
13	1.72	2.21	3.93	0.93	1.28	m	M1
14	1.71	2.21	3.92	0.93	1.29	m	M1
15	1.60	2.29	3.89	0.92	1.43	m	M1
16	1.60	2.29	3.89	0.93	1.43	m	M1
17	1.70	2.07	3.77	0.90	1.22	m	M1
18	1.67	2.07	3.74	0.89	1.24	m	M1
19	1.69	2.04	3.73	0.89	1.22	m	M1
20	1.68	2.00	3.68	0.88	1.19	m	M1
21	1.65	1.97	3.62	0.86	1.19	m	M1
22	1.64	1.92	3.56	0.85	1.17	m	M1
23	1.49	1.74	3.43	0.82	1.167	m	M1
24	1.43	1.72	3.15	0.75	1.2	m	M1

Length of l short arm chromosome (LS), length of long arm chromosome (LL), length of total chromosome (LT), arm ratio (AR), relative length (RL), centromeric index (CI) , arm ratio (AR), centromere type (CT), chromosome length type (CLT) 2n (diploid) = 48

Supplementary Table 3. Nine components of *A. oxyphylla* as determined by RP-HPLC

Production regions	地点	Nootkatone	Yakuchinone A	Yakuchinone B	Oxyphyllacinol	Tectochrysin	Izalpinin	Chrysin	Kaempferide	apigenin-7,4'-dimethylether
1#-01	DZ	685	1.2025	0.001315	0.22375	0.00109	<0	0.028	0.04525	0.13125
1#-02	DF	775	0.195	<0	<0	<0	<0	0.01025	<0	0.12375
1#-03	BS	467.5	0.56	<0	<0	0.000562	<0	0.05325	<0	0.12525
1#-04	QZ	505	0.72	0.000855	<0	0.017925	<0	0.0715	0.00325	0.12825
1#-05	WZS	158.25	0.64	<0	<0	0.015	<0	0.049	0.03875	0.126
1#-06	BT	550	0.475	0.0010075	<0	0.0032	<0	0.0043	<0	0.123
1#-07	WN	537.5	0.57	0.000056	<0	0.00675	<0	0.06775	0.009525	0.12825
1#-08	HK	262.5	0.3125	<0	<0	0.02875	<0	0.025	0.012925	0.13125
1#-09	FCG	98.5	0.565	<0	<0	0.003625	<0	0.02425	0.02725	0.129
1#-10	NN	131.25	0.22925	<0	<0	<0	<0	0.006975	0.000795	0.12525
1#-11	RX	345	0.485	<0	<0	<0	<0	0.06025	0.003075	0.12375
1#-12	XY	425	0.203	0.000068	<0	<0	<0	0.0149	0.02875	0.123
1#-13	GZ	477.5	0.24925	<0	<0	<0	<0	0.02095	<0	0.12375
1#-14	YC	312.5	0.42	<0	<0	0.0295	<0	0.026	0.017475	0.132
1#-15	XSBN	485	0.315	<0	<0	<0	<0	0.013275	<0	0.132
1#-16	ZP	122.5	0.725	0.0017	<0	0.019575	<0	0.06675	<0	0.14375
1#-17	FZ	97.75	0.24275	<0	<0	<0	<0	0.0172	0.00193	0.12225

Supplementary Table 4. The total variance of the 9 components explained

Components	Total	Initial eigenvalue variance %	Initial eigenvalue Accumulation %	Extract sum of squares and load Sum	Extract sum of squares and load Variance %	Extract sum of squares and load Accumulation %
1Nootkatone（NOOT）	3.592	39.906	39.906	3.592	39.906	39.906
2 Kaempferide (KAEM	2.119	23.547	63.453	2.119	23.547	63.453
3 Yakuchinone B (YB)	1.467	16.298	79.751	1.467	16.298	79.751
4 Oxyphyllacinol (OXYP)	0.864	9.602	89.353	-	-	-
5 Tectochrysin（TECT）	0.430	4.782	94.135	-	-	-
6 Izalpinin (IZAL)	0.244	2.714	96.850	-	-	-
7 Chrysin (CHRY)	0.153	1.702	98.552	-	-	-
8)Yakuchinone A（YA）	0.080	0.888	99.440	-	-	-
9 apigenin-7,4'-dimethylether (APIG)	0.050	0.560	100.000	-	-	-

Supplementary Table 5. Results of component matrix

compounds	Component 1	Component 2	Component 3
NOOT	.078	.766	-.453
KAEMP	.685	.551	.305
YB	.836	.301	-.108
OXYP	.691	.532	.351
TECTO	.815	-.145	-.138
IZAL	.707	-.488	-.208
CHRY	-.054	-.014	.951
YA	.415	-.647	.220
APIG	.775	-.421	-.138

Supplementary Table 6. Comprehensive score of 9 components content in different regions

Regions	Y1	Y2	Y3	Y	Ranking
DZ	5.5	0.82	-1.26	2.18	1
DF	0.65	1.95	-1.06	0.55	4
BS	0.8	-0.38	0.45	0.3	5
QZ	2.43	-1.01	1.75	1.02	3
WZS	-0.7	-0.49	-0.14	-0.42	10
BT	0.42	2.04	2.32	1.03	2
WN	-1.24	1.39	1.12	0.01	7
HK	-1.61	0.41	-0.55	-0.64	14
FCG	-2.22	0.82	0.12	-0.67	15
NN	-1.05	-0.82	-1.78	-0.9	16
RX	-0.8	-0.58	0.01	-0.45	11
XY	1.74	-2.11	-1.23	0	8
GZ	-0.82	-0.94	0.63	-0.45	11
YC	-0.27	-0.42	0.09	-0.19	9
XSBN	-0.09	0.27	1.33	0.24	6
ZP	-2	0.24	-1.92	-1.06	17
FZ	-0.74	-1.18	0.13	-0.55	13

Supplementary Table 7. Primers used in this study for qRT-PCR analysis.

Primer	Sequence (5' to 3')
Cluster-28762.29015	CTGACGACGTTGAACACGG
Cluster-28762.29015	TTCCTGACGACGTTGAACAC
Cluster-28762.51249	TGGAAGGTGAAGATGGCTGATT
Cluster-28762.51249	GTGGAAGGTGAAGATGGCTGAT
Cluster-28762.16902	CCAGTGCGATGAAAACAGCG
Cluster-28762.16902	GTAGCTGGTGCTCAGGATCG
Cluster-28762.27379	ACTACCCTCTACTCCACCCA
Cluster-28762.45651	GTACGCAGCATTTGTCCATCC
Cluster-28762.45651	ACGCAGCATTTGTCCATCC
Cluster-28762.11998	CCTACCGCCATCTGTGTCTC
Cluster-28762.11998	GCCTACCGCCATCTGTGTCT
Cluster-28762.52286	TTCGCTTCTGCCTCCGTTC
Cluster-28762.52286	TTCTTCGCTTCTGCCTCCGT
Cluster-28762.4998	GCACGCCCCGATCTTGA
Cluster-28762.4998	AGCTTGAGCTTGATGGTGGT
Cluster-28762.4360	CGACTTCGCCGAGTACCAC
Cluster-28762.4360	AACGACTTCGCCGAGTACCA
Cluster-28762.2807	GTGTTCTCCTACTGCTTGCC
Cluster-28762.2807	ACTTTTAGGGTTAGGTCGCGG
Cluster-28762.51240	ACGACGAGTTCGAGGTGCAA
Cluster-28762.51240	CGCTGGAGGCCTCAACGATA

Supplementary Table 8. Estimation of *A. oxyphylla* genome size based on flow cyrtometry statistics.

species	Measured value	CV	Mean value	Genome size(Mb)
M. esculenta 1	6530	3.93	6539.25	760
M. esculenta 2	6568	3.72		
M. esculenta 3	6542	3.71		
M. esculenta 4	6517	4.4		
S. lycopersicum 1	8190	3.94	8180	960
S. lycopersicum 2	8235	3.55		
S. lycopersicum 3	8223	3.8		
S. lycopersicum 4	8073	3.97		
A. oxyphylla 1	15840	4.5	15682.25	1822.61
A. oxyphylla 2	15758	3.49		
A. oxyphylla 3	15608	4.09		1840.46
A. oxyphylla 4	15523	3.89		

Supplementary Table 9. Estimation of *A. oxyphylla* genome size based on 17 K-mer statistics.

Clean data (bp)	Q30(%)	Sequencing depth (X)	GC content (%)
112,774,962,900	91.19	54.02	39.45
K-mer	Main peak depth of K-mer	Estimated genome size (Mb)	Heterozygote rate (%)
17	40	2,144.66	0.99

Supplementary Table 10. PacBio circular consensus sequencing (CCS) sequencing and Hi-C technology parameters

Reads Type	Total bases (Gb)	Total reads	Reads N50 (Bp)	Mean Length (bp)	Sequence coverage (X)	Function
PacBio	80.10	29149513	2360	2122	54.02	assembly
Illumina	115.86		-		37.35	

Supplementary Table 11. Hi-C assisted genome assembly results

Sample ID	Contig length	Scaffold length	Contig number	Scaffold number	Total length
Total	2,085,722,329	2,085,726,029	572	535	-
Max	107,591,051	113,791,837	-	-	-
Number>=2000	-	-	572	535	-
N50	76,960,141	83,048,840	12	11	-
place	-	-	-	24	2,004,011,653
unplace	-	-	-	511	81,714,376
total			-	535	2,085,726,029
Anchored rate			96.08%		

Supplementary Table 12. Evaluation of Benchmarking Universal Single-Copy Orthologs (BUSCO) and GeneSpace Coverage Using Core Eukaryotic Gene Mapping Approach (CEGMA) in *A. oxyphylla* genome.

Type	Number	Percent (%)
Complete BUSCOs (C)	1528	94.6
Complete and single-copy BUSCOs (S)	1432	88.7
Complete and duplicated BUSCOs (D)	96	5.9
Fragmented BUSCOs (F)	31	1.9
Missing BUSCOs (M)	55	3.5
Total BUSCO groups searched (n)	1614	
Number of 248 highly conserved CEGs present	235	94.76

Supplementary Table 13. Coverage statistics of small fragment reads on *A. oxyphylla* genome

	parameter	Perceperntage or number
Reads	Mapping rate (%)	99.30
Genome	Average sequencing depth	45.88
	Coverage (%)	99.95
	Coverage at least 4X (%)	99.91
	Coverage at least 10X (%)	99.77
	Coverage at least 20X (%)	97.03

Supplementary Table14. statistics of SNP in *A. oxyphylla* genome

	Number	Percentage
All SNP	9,997,323	0.481601%
Heterozygosis SNP	9,996,563	0.481565%
Homology SNP	760	3.7e-05%

Table15. Statistics of repeat contents in *A. oxyphylla* genome

	Denovo+Repbase		TE Proteins		Combined TEs	
	Length(bp)	% in Genome	Length(bp)	% in Genome	Length(bp)	% in Genome
DNA	46,652,940	2.24	4,836,193	0.23	50,987,170	2.44
LINE	18,730,109	0.90	3,862,329	0.19	21,697,434	1.04
SINE	307,670	0.01	0	0	307,670	0.01
LTR	1,158,421,691	55.54	457,977,580	21.96	1,286,859,992	61.70
Unknown	826,558,413	39.63	0	0	826,558,413	39.63
Total	1,821,377,876	87.33	466,674,855	22.37	1,831,597,484	87.82

Supplementary Table 16. Basic statistical results of gene structure of proximal species

Species	Number	Average transcript length(bp)	Average CDS length(bp)	Average exons per gene	Average exon length(bp)	Average intron length(bp)
A. oxyphylla	38,178	5,597.69	1,116.05	4.49	248.53	1,283.90
E. ventricosum	58,438	1,772.99	676.22	2.39	283.25	790.54
Z. officinale	101,895	5,984.85	1,364.08	5.93	230.09	937.58
M. balbisiana	35,148	4,441.90	1,144.37	5.06	226.29	812.79
O. sativa	42,373	2,201.70	991.09	3.90	254.40	418.05
M. acuminata	36,519	3,593.53	1,038.40	5.41	191.94	579.39
A.thaliana	35,386	1,947.90	1,230.62	5.57	220.87	156.90

Supplementary Table 17. Gene function annotation statistical results of *A. oxyphylla*

	Number	Percent(%)
Total	38,178	-
Swissprot	28,805	75.40
Nr	35,840	93.90
KEGG	28,140	73.70
InterPro	30,957	81.10
GO	20,840	54.60
Pfam	27,990	73.30
Annotated	35,917	94.10
Unannotated	2,261	5.90

Supplementary Table 18. Statistical results of non-coding RNA

Copy number	Average length(bp)	Total length(bp)	% of genome
534	125.99	67,279	0.003226
3,928	74.49	292,602	0.014029
9,249	351.16	3,247,879	0.16
1,756	1,318.98	2,316,121	0.11
5,002	120.10	600,724	0.028802
1,264	155.33	196,340	0.009414
1,227	109.78	134,694	0.006458
10,423	107.19	1,117,232	0.053566
10,256	106.69	1,094,210	0.052462
74	139.80	10,345	0.000496
93	136.31	12,677	0.000608

Supplementary Table 19. Gene family analysis of *A. oxyphylla* and other 9 species

Item	*A.comosu*	*A.oxyp*	*A.thalia*	*C.alisma*	*M.acumin*	*M.balbis*	*O.sativa*	*S.bicolo*	*W.villos*	*Z. offici*
Number of genes	27,024	35,315	27,381	57,492	29,345	33,021	55,973	34,129	42,578	37,770
Number of genes in orthogroups	20,690	30,717	23,191	48,905	28,478	26,501	42,100	27,218	37,471	32,923
Number of unassigned genes	6,334	4,598	4,190	8,587	867	6,520	13,873	6,911	5,107	4,847
Percentage of genes in orthogroups	76.6	87	84.7	85.1	97	80.3	75.2	79.8	88	87.2
Percentage of unassigned genes	23.4	13	15.3	14.9	3	19.7	24.8	20.2	12	12.8
Number of orthogroups containing species	13,656	15,972	12,898	15,978	14,491	14,424	18,111	16,372	16,767	14,264
Percentage of orthogroups containing species	40.7	47.6	38.5	47.6	43.2	43	54	48.8	50	42.5
Number of species-specific orthogroups	892	440	1,258	1,742	49	315	2,446	856	860	967
Number of genes in species-specific orthogroups	3,067	1,747	5,142	19,740	171	1,210	17,267	2,700	4,114	8,249
Percentage of genes in species-specific orthogroups	11.3	4.9	18.8	34.3	0.6	3.7	30.8	7.9	9.7	21.8

Supplementary Table 20. Transcriptome sequencing results of *A. oxyphylla* from 4 regi

Sample number	Region	Clean reads pair	Clean bases(bp)	Mapping rate (%)	Q30(%)
A1	Danzhou	26716971	7.99	80.82	93.60
A2	Danzhou	30675380	9.17	88.33	93.61
A3	Danzhou	29094268	8.70	90.46	93.68
A4	Baoting	27552390	8.24	69.62	92.74
A5	Baoting	30600398	9.15	73.51	93.59
A6	Baoting	26676795	7.98	76.47	93.66
B1	Nanning	28097382	8.40	94.45	93.51
B2	Nanning	29942426	8.95	93.45	93.63
B3	Nanning	31107308	9.30	94.26	93.48
B4	Zhangpu	26731086	7.99	91.19	93.37
B5	Zhangpu	31400225	9.39	91.69	93.63
B6	Zhangpu	28855674	8.62	91.62	93.76
